# The effects of time-restricted eating and Ramadan fasting on gut microbiota composition: a systematic review of human and animal studies

**DOI:** 10.1093/nutrit/nuad093

**Published:** 2023-08-01

**Authors:** Joanna Maria Pieczyńska-Zając, Anna Malinowska, Karolina Łagowska, Natalia Leciejewska, Joanna Bajerska

**Affiliations:** Department of Human Nutrition and Dietetics, Poznań University of Life Sciences, Poznań, Poland; Laboratory of Microbiology, Wageningen University and Research, Wageningen, The Netherlands; Department of Human Nutrition and Dietetics, Poznań University of Life Sciences, Poznań, Poland; Department of Physiology, Biochemistry, and Biostructure of Animals, Poznań University of Life Sciences, Poznań, Poland; Department of Human Nutrition and Dietetics, Poznań University of Life Sciences, Poznań, Poland

**Keywords:** chrononutrition, fasting, gut microbiota, host health, microbial diversity

## Abstract

**Context:**

It is well known that the microbiome undergoes cyclical diurnal rhythms. It has thus been hypothesized that meal timing may affect gut microbial composition, function, and host health.

**Objective:**

This review aims to examine the effects of time-restricted eating (TRE) and Ramadan fasting (RF) on the composition of the gut microbiota in animal and human studies. The associations between composition of microbiota and host metabolic parameters are also examined.

**Data Sources:**

A search was performed on the PubMed, Cochrane, Scopus, and Web of Science databases up to December 31, 2022. The search strategy was performed using the Medical Subject Heading (MeSH) terms “intermittent fasting” and “gastrointestinal microbiome” and the key words “Ramadan fasting” and “microbes.”

**Data Extraction:**

Seven human studies (4 TRE and 3 RF) and 9 animal studies (7 TRE, 2 RF-like) were retrieved.

**Data Analysis:**

TRE and RF in human studies lead to an increase in gut microbial community alpha-diversity. In animal studies (both TRE and RF-like), fasting is not associated with improved alpha-diversity, but enhancement of microbial fluctuation is observed, compared with high-fat diet ad libitum groups. Within Firmicutes and Bacteroidetes phyla, no specific direction of changes resulting from fasting are observed in both animals and human. After TRE or RF, a greater abundance of the *Faecalibacterium* genus is observed in human studies; changes in *Lactobacillus* abundance are found in animal studies; and increases in *Akkermansia* are seen both in humans and in animals fed a feed-pellet diet. Only 2 human studies show a beneficial correlation between microbiota changes and host metabolic (HDL cholesterol) or anthropometric parameters (body mass index).

**Conclusions:**

These findings support the importance of both regimens in improving the gut microbiota composition. However, based on results of animal studies, it can be suggested that diet remains the essential factor in forming the microbiota’s environment.

**Systematic Review Registration:**

PROSPERO registration no. CRD42021278918.

## INTRODUCTION

Circadian rhythms represent an endogenous time-keeping system that regulates and synchronizes behavior, physiology, and metabolism with external cues known as zeitgebers, thus establishing homeostasis.^1^ The light–dark cycle is the most important zeitgeber, but other stimuli such as temperature and the presence of food can also act as zeitgebers.^2^ Circadian rhythms are regulated by a master clock located in the suprachiasmatic nucleus of the hypothalamus,^3^ as well as by peripheral clocks in other tissues, including the liver, muscle, adipose tissue, and even the gut. At the molecular level, the circadian clock consists of multiple sets of transcription factors that regulate gene expression, operating in a series of feedback loops.^4^

The gut microbiota provides many benefits to the host, biosynthesizing vitamins and essential amino acids and generating important metabolic byproducts, including short-chain fatty acids, such as butyrate, propionate, and acetate, that act as major energy sources for intestinal epithelial cells, and which may therefore strengthen the mucosal barrier.^5^ Diet is a key factor for gut microbiota composition and metabolism, and several studies have investigated the effects of different dietary components, including dietary fiber, on the gut microbiota.^6^^,^^7^ On the other hand, a high-fat diet (HFD) has been shown to adversely alter the composition of the gut microbiota, reducing microbial diversity and depleting the abundance of beneficial bacteria, including *Bifidobacterium* and *Akkermansia*,^8^ which are believed to have beneficial effects on body weight and on carbohydrate metabolism parameters.^9^

It is known that the microbiome undergoes cyclical diurnal rhythms.^10^ The greatest peak in bacteria of the Bacteroidetes and Veruccomicrobia phyla can be observed in rodents during feed-deprivation periods. The number of these bacteria gradually decreases with the approach of the feeding period, and bacteria of the Firmicutes phylum instead dominate.^10^ This is a particularly important aspect that should be taken into account in the methodology of research on the composition of the microbiota, because these cyclical changes can be observed only in the intestinal contents collected during the circadian termination of rodents.^10^^,^^11^ Therefore, the assessment of animal microbiota directly from the intestinal contents seems to be more accurate than from the feces, both human and animal, which allows the observation of changes in only 1, often impossible to determine, time point.

It is also hypothesized that meal timing may also affect the gut microbiome, with implications for host health.^5^ One dietary regimen that may affect peripheral oscillations is time-restricted eating (TRE), a pattern where food intake is restricted to certain hours of the day (most often an 8-h period), with no limitation on nutrient quality or quantity.^11^ One form of TRE is Ramadan fasting (RF), a regimen that is common among Muslims.^12^ Those practicing RF fast from sunrise to sunset, eating 2 or 3 meals after sunset. However, during Ramadan, there is also a change in the quality of the diet, with increased consumption of cakes, sweetened drinks, vegetables, and dried fruits, and decreased consumption of fats, dairy products, eggs, and cereal products.^13^ Meals are mainly consumed during the day in TRE, but in RF they are mainly consumed at night, which may have an effect on gut microbiota composition and metabolic health of the host.

Considering that both dietary regimens may be significant modulators of health and microbiota diversity, the aim of this systematic review is to summarize the effects of the TRE and RF regimens on the composition of the gut microbiota in both animal and human studies. Extensive research using both animal models^10^^,^^11^^,^^14^^,^^15^^,^^16^ and humans^17^^,^^18^ demonstrates that both TRE and RF yield beneficial changes in the metabolic parameters associated with obesity; for this reason, the aim was also to investigate whether the changes in these host metabolic parameters are associated with changes in the composition of the gut microbiota.

## METHODS

### Study eligibility

This systematic review was registered in the International Prospective Register of Systematic Reviews (CRD42021278918) and was conducted in line with the principles of the Preferred Reporting Items for Systematic Reviews and Meta-Analyses (PRISMA) statement (Table S4*in theSupporting Information online*).

### Search strategy and inclusion/exclusion criteria

A search was performed by J.M.P.-Z. and J.B. on the PubMed, Cochrane, Scopus, and Web of Science databases from January 1, 2005, up to December 31, 2022. The search strategy was performed using both Medical Subject Heading (MeSH) terms and key words. The search for TRE used the terms “intermittent fasting” (a MeSH term) OR “ramadan fasting” (a key word). For gut microbiota, the search was carried out using the terms “gastrointestinal microbiome” (a MeSH term) OR “microbes” (a keyword).

The Population, Intervention, Comparison, Outcomes, and Study (PICOS) design criteria were used to identify all of the quantitative research studies for the present literature review (Table 1). Any interventional and observational studies that met the following eligibility criteria were included: (1) study participants were humans aged 18–65 years or rodents older than 6 weeks who underwent TRE or RF for at least 3 weeks; outcomes included changes in the composition of the gut microbiota at different taxonomic levels (assessed by 16S rRNA) and its alpha- and beta-diversity. Selected associations between the composition of the gut microbiota and host metabolic parameters or body weight (secondary outcomes) were also evaluated. Systematic reviews, case reports, articles written in a language other than English, and papers in which a treatment arm (other than TRE/RF) included exercise, calorie restriction, or weight-loss supplementation were excluded.

**Table 1:** PICOS (Population, Intervention, Comparison, Outcomes, and Study) criteria for inclusion of studies

Parameter	Description
Population	Humans aged 18–65 years Rodents older than 6 weeks
Intervention	Time-restricted eating or Ramadan fasting regimen for at least 3 weeks
Comparison	Nonfasting/ad libitum groups Baseline parameters
Outcomes	Changes in the composition of gut microbiota at different taxonomic levels (assessed by 16S rRNA) Alpha- and beta-diversity Associations between the composition of the gut microbiota and host metabolic parameters or body weight

**Figure 1:**
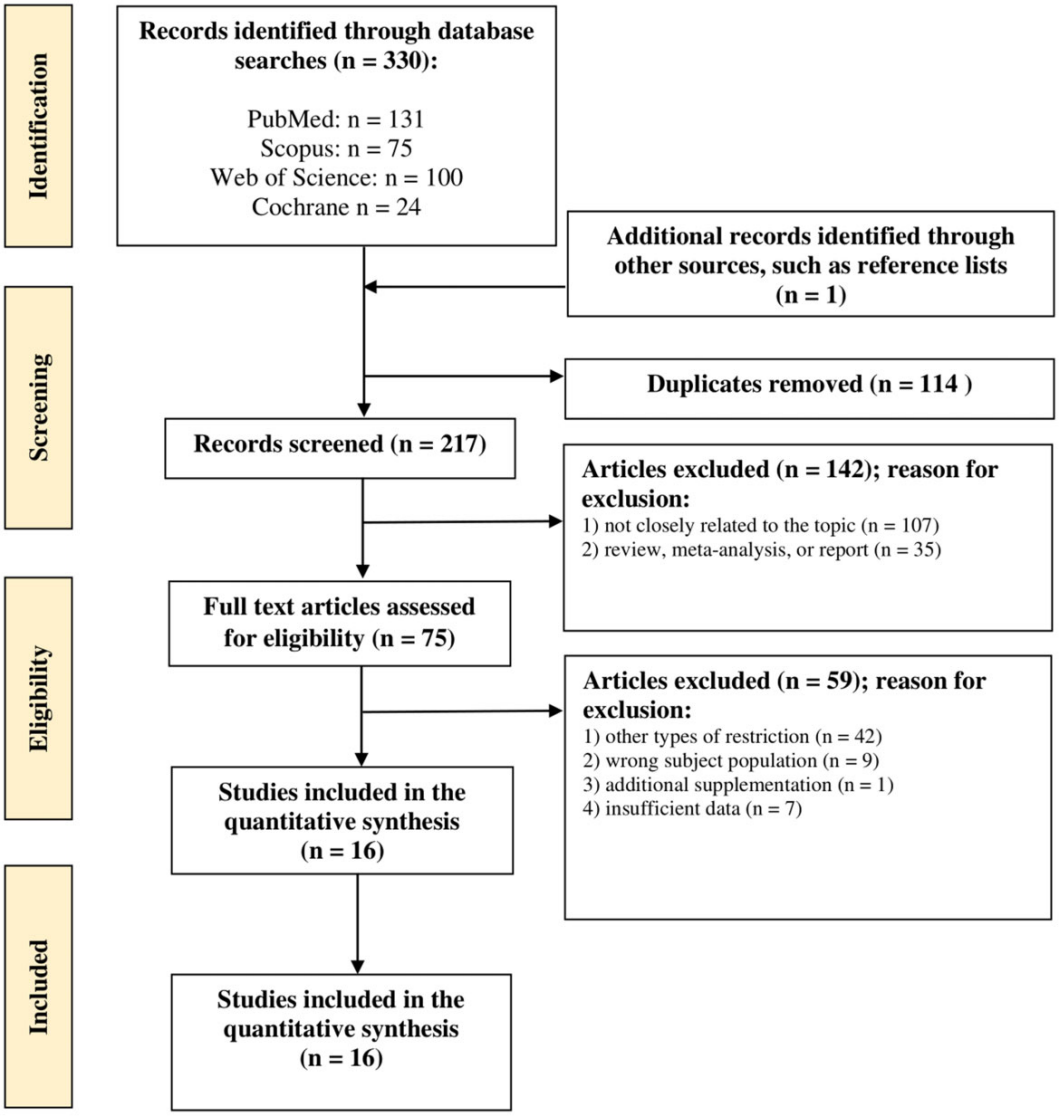
Flow diagram showing the study selection process.

The search results from all of the databases were collected in the Mendeley tool (Mendeley Desktop Version 1.19.8), where duplicates were removed. A 2-phase search strategy was subsequently used by 2 independent reviewers (J.M.P.-Z. and J.B.) up to December 31, 2022. In phase 1, the eligibility of each study was assessed on the basis of its title and abstract. Studies that had questionable suitability were provisionally included, with a final decision made in phase 2. In phase 2, full articles were retrieved and assessed against the eligibility criteria. Reference lists of original and review articles were screened to ensure that all relevant studies had been included. Any disagreement over the eligibility of an article for this study was resolved through discussion with K.Ł., A.M., and N.L. The search strategy is summarized in Fig. 1.

### Data extraction

The following data were extracted from the animal studies: author, type of animal model, number of animals and their age, type of intervention, control conditions, intervention diet, and duration of the study. The following outcomes were extracted from the animal studies: type of material (colonic or fecal) taken to test the composition of the microbiota, the variable gene region selected for gene sequencing, abundance of microbial taxa at the phylum and genus level, the alpha-diversity and beta-diversity parameters, as well as other study findings, such as associations between changes in the microbiota and host metabolic markers.

The following data were extracted from the human studies: author, study design, number of participants, age (years), type of intervention, control conditions, and duration of the study. The following outcomes were extracted from the human studies: type of material (feces), the variable gene region selected for gene sequencing, abundance of microbial taxa at the phylum and genus level, alpha-diversity and beta-diversity parameters, and other study findings, such as associations between changes in microbiota and host metabolic markers.

Any disputes regarding the appropriateness of including or excluding a given study were resolved by discussion between the authors.

### Quality assessment

For the rodent model studies, the Systematic Review Centre for Laboratory Animal Experimentation (SYRCLE) risk-of-bias assessment tool was used.^19^ For the human studies, the Quality Assessment Tool for Observational Cohort and Cross-Sectional Studies, the Quality Assessment Tool for Before–After (Pre–Post) Studies With No Control Group, and the Quality Assessment of Controlled Intervention Studies from the National Institutes of Health (NIH) National Heart, Lung, and Blood Institute^20^ were used.

## RESULTS

Due to the nature of the data, the limited number of studies, the large heterogeneity they displayed, the range of designs used, the various methodologies for determining microbiota, and various ways of presenting the results, it was decided to systematically summarize the current evidence, rather than performing a quantitative meta-analysis. Differences between groups and changes within the study group before and after the intervention are reported. In order to unify the text, the abbreviation TRE is used in reference to both the human and animal studies.

### Reviewed studies

Overall, 331 articles were identified and the final analysis included 7 human studies and 9 animal studies from 16 papers (Fig. 1). The quality of all included studies are rated “good” or “fair.” None of the studies were of “poor” quality. All animal studies were assessed as “good.” In turn, 2 of the human studies^21^^,^^22^ were rated as “fair.” One of them, which was cross-sectional, gave no specific inclusion or exclusion criteria and no exact recruitment period.^22^ The other study, which was a randomized controlled trial, gave no specific randomization method and did not calculate a minimum sample size, despite this being necessary in this type of study.^21^

It is worth mentioning that, due to the nature of TRE and RF, it is impossible to blind the research participants and the animal caregivers. For this reason, blinding is not taken into account when assessing the quality of studies. Tables S1 and S2 (*see the Supporting Information online*) present the full details.

The publication dates were between January 6, 2020,^21^ and February 22, 2022,^23^ for the human studies and between December 2, 2014,^10^ and July 5, 2022,^24^ for the animal studies. The duration of TRE or RF differed between the studies, from 25 days^21^^,^^22^ to 12 weeks^25^ in the human studies and from 4 weeks^15^ to 48 weeks^26^ in the animal studies.

Of the 9 rodent studies, 7 were conducted in mice—specifically Kunming mice (2 studies),^11^^,^^14^ C57BL/6J mice (5 studies),^10^^,^^15^^,^^16^^,^^24^^,^^27^ and BALB/c mice (1 study).^28^ One study was conducted in DPP-IV–Fischer rats.^26^ Their details, housing, and diet treatments are presented in Table 2.^10^^,^^11^^,^^14^^,^^15^^,^^16^^,^^24^^,^^26^^,^^27^^,^^28^ All rodents were 6 weeks of age or older to ensure maturity. Each of the articles used an 8-hour eating window and a 16-hour fasting/feed-deprivation period, with the single exception of Li et al^27^ who used not only the traditional 8/16 regimen but also a 12/12 regimen (12 h of access to feed and 12 h of feed deprivation) and a 4/20 regimen (4 h of access to feed and 20 h of feed deprivation). Moreover, in 2 animal studies,^15^^,^^28^ the eating window was during the resting phase of the rodents, which more closely mimics the Ramadan pattern.^15^ The most common diets were feed-pellet diets and HFDs, although in 1 study a lithogenic diet was used.^15^ The control for the TRE or Ramadan-like fasting was the same, or feed-pellet diet given ad libitum (AL).

**Table 2:** Details of the animal studies

Source	Animal models	Model	n	Specimens	Variable gene region for gene sequencing	Age	Dietary regimen	Intervention diet	Control	Duration	Comparison	Outcomes		
												Alpha-diversity	Beta-diversity	Taxonomic composition
Zarrinpar et al, 2014^10^	C57BL/6J mice	Male	72	Cecal contents	V1-V3	10-wk-old	TRE 8/16	HFD	HFD AL; CD AL	8 wk	TRE (HFD) vs AL (HFD) TRE (HFD) vs AL (CD)	X	X	X
Ye et al, 2020^11^	Kunming mice	Male	60	Rectal contents	V3-V4	8-wk-old	TRE 8/16	HFD	HFD AL; CD AL	8 wk	TRE (HFD) vs AL (HFD) TRE (HFD) vs AL (CD)	X	X	X
He et al, 2021^15^	C57BL/6J mice	Male	60	Cecal contents	NR	6-wk-old	RF-like 12/12	LD	LD AL	4 wk	RF-like (LD) vs AL (LD)		X	X
Machado et al, 2022^24^	C57BL/6J mice	Male	54	Ileal/cecal contents	V1-V3 (cecal) V3-V4 (ileal)	8-wk-old	TRE 8/16	HFD	HFD AL; CD AL	8 wk	TRE (HFD) vs AL (HFD) TRE (HFD) vs AL (CD)	X	X	X
Hu et al, 2018^14^	Kunming mice	Male	40	Cecal contents	V1-V3	9-wk-old	TRE 8/16	CD	CD AL	2 mo	TRE (CD) vs AL (CD)	X	X	X
Li et al, 2020^27^	C57BL/6Jlvri mice	Male	15	Feces	V3-V4	7-wk-old	TRE 8/16	CD	CD AL	1 mo	TRE (CD) vs AL (CD)	X	X	X
	C57BL/6Jlvri mice	Male	15	Feces	V3-V4	7-wk-old	TRE 12/12	CD	CD AL	1 mo	TRE (CD) vs AL (CD)	X	X	X
	C57BL/6Jlvri mice	Male	15	Feces	V3-V4	7-wk-old	TRE 4/20	CD	CD AL	1 mo	TRE (CD) vs AL (CD)	X	X	X
van der Merwe et al, 2020^16^	C57BL/6J mice	Male	43	Feces/cecal contents	NR	12-wk-old	TRE 8/16	HFD	HFD AL	6 wk	TRE (HFD) vs AL (HFD)	X	X	X
Palomba, 2021^26^	DPP-IV–Fischer rats	Male	16	Feces	V4	8-wk-old	TRE 8/16	CD	CD AL	48 wk	TRE (CD) vs AL (CD)			X
Su et al, 2022^28^	BALB/c mice	Male	14	Feces	V3-V4	6-wk-old	RF-like 8/16	CD	CD AL	1 mo	RF-like (CD) vs AL (CD)	X	X	X

*Abbreviations*: AL, ad libitum; CD, chow (feed-pellet) diet; HFD, high-fat diet; LD, lithogenic diet containing 1.25% cholesterol and 0.5% cholic acid; NR, not reported; RF-like, Ramadan-like fasting; TRE, time-restricted eating; X, data presented; 8/16, 8-hour eating window, 16-hour fasting; 12/12, 12-hour eating window, 12-hour fasting; 4/20, 4-hour eating window, 20-hour fasting.

Of the 7 human studies, 2 were randomized controlled trials,^21^^,^^23^ 1 study had a quasi-experimental design,^25^ and 1 study was cross-sectional.^22^ The 3 RF human studies all had an observational design.^17^^,^^18^^,^^29^ Six studies enrolled apparently healthy individuals,^17^^,^^18^^,^^21^^,^^22^^,^^23^^,^^29^ while 1 study enlisted patients with obesity.^25^ All participants were aged from 18 to 56 years (Table 3^17^^,^^18^^,^^21^^,^^22^^,^^23^^,^^25^^,^^29^). Four studies^21^^,^^22^^,^^23^^,^^25^ explored the effects of TRE (with an 8-h eating window and a 16-h fast vs a normal diet), while in 1 of these studies TRE was divided into early and mid-day.^23^ The other 3 studies were undertaken during the month of Ramadan with a 7-hour or 8-hour eating window during the night^17^^,^^18^^,^^29^; in the study by Su et al,^18^ 2 study groups were included—one younger and one middle-aged—and were analyzed separately. In addition, the results of the young cohort were analyzed in relation to the baseline parameters, while those of the middle-aged cohort were compared with the results of a nonfasting control group.^18^

**Table 3:** Details of the human studies

Source	Study design	Participants	n	Age, y	Specimens	Intervention	Control	Duration	Comparison	Variable gene region for gene sequencing	Outcomes		
											Alpha-diversity	Beta-diversity	Taxonomic composition
Time-restricted eating													
Gabel et al, 2020^25^	Quasi-experiment	Obese adults	14	25–65	Feces	TRE 8/16	—	12 wk	Post vs pre	V4	X		X
Zeb et al, 2020^22^	Cross-sectional	Healthy males	30	18–38	Feces	TRE 8/16	AL	25 d	TRE vs AL	V3-V4	X	X	X
Zeb et al, 2020^21^	RCT	Healthy males	80	> 18	Feces	TRE 8/16	AL	25 d	TRE vs AL	V1-V3	X	X	X
Xie et al, 2022^23^	RCT	Healthy adults	82	eTRE: 28.68 ± 9.707 mTRE: 31.08 ± 8.438 AL: 33.57 ± 11.6	Feces	eTRE 8/16 mTRE 8/16	AL	5 wk	TRE vs AL	V3-V4	X		X
Ramadan fasting													
Ozkul et al, 2020^17^	Observational	Healthy adults	9	31–56 (45.0 ± 9.7)	Feces	RF 7/17	—	29 d	Post vs pre	V4	X	X	X
Su et al, 2021^18^	Observational	Healthy males (young cohort)	30	18.63 ± 1.75	Feces	RF 8/16	—	30 d	Post vs pre	V3-V4	X	X	X
	Observational	Healthy adults (middle-aged cohort)	37	NF: 42.6 ± 7.9; F: 39.9 ± 6.4	Feces	RF 8/16	AL	30 d	RF vs AL	V3-V4	X	X	X
Ali et al, 2021^29^	Observational	Healthy adults	34	18–40	Feces	RF	—	1 mo	Post vs pre	V3-V4	X	X	X

*Abbreviations*: AL, ad libitum; eTRE, early TRE; F, fasting; mTRE, mid-day TRE; NF, nonfasting; NR, not reported; RCT, randomized controlled trial; RF, Ramadan fasting; TRE, time-restricted eating; X, data presented; 8/16, 8-hour eating window, 16-hour fasting; 7/17, 7-hour eating window, 17-hour fasting.

### Method of microbiome assessment

Fecal specimens from all of the human studies (n = 7) were analyzed for gut microbiota composition. Of the 9 rodent studies, 5 used cecal, ileal, or rectal contents^10^^,^^11^^,^^14^^,^^15^^,^^24^; 3 studies used fecal material^26–28^; and 1 study used both types.^16^ The 16S rRNA amplicon sequencing method was used in both human and animal studies. In the animal studies, 3 of them use the V3-V4 hypervariable region,^11^^,^^27^^,^^28^ 2 studies used the V1-V3 region,^10^^,^^14^ 1 study used the V4 region,^26^ and 1 study used the V1-V3 region for cecal content and the V3-V4 region for ileal contents.^24^ The rest do not specify a region.^15^^,^^16^ In the case of human studies, 4 studies used the V3-V4 region,^22^^,^^23^^,^^28^^,^^29^ 2 studies used the V4 region,^17^^,^^25^ and 1 study used the V1-V3 region.^21^

### Outcomes of microbiome assessment

The synthesized results from the animal and human studies report on 2 major taxonomic levels, P (phylum) and G (genus), of bacterial taxa, although not all of these are analyzed in each group and in each study.^11^^,^^15^^,^^16^^,^^18^^,^^18^^,^^23–27^Table 4^10^^,^^11^^,^^14^^,^^15^^,^^16^^,^^24^^,^^26^^,^^27^^,^^28^ presents the effects of TRE on the composition, alpha-diversity, and beta-diversity of microbiota in the animal studies. Table 5^17^^,^^18^^,^^21^^,^^22^^,^^23^^,^^25^^,^^29^ presents the effects of TRE and RF on the composition, alpha-diversity, and beta-diversity of microbiota in the human studies. Due to the large number of results from the animal studies, Table 4 presents only those changes that concerned phyla or genera repeated in most studies. The full results are presented in Table S3 (*see the Supporting Information online*).

**Table 4:** Effects of TRE regimen at the phylum and genus level, and in alpha- and beta-diversity in animal studies

Source	Source of microbiota	Comparison	Phyla: differences between groups	Phyla: differences between phases		Genera: differences between groups	Genera: differences between phases		Alpha-diversity	Beta-diversity
Circadian termination										
Zarrinpar et al, 2014^10^	Cecum sample; every 4 h over 24 h (ZT1, 5, 9, 13, 17, 21)	HFD TRE vs HFD AL	↔Firmicutes ↔Bacteroidetes ↔Veruccomicrobia	NR		*↗ Oscillibacter* (0.40 ± 0.08% vs 0.13 ± 0.04%) *↘ Lactobacillus* (0.97 ± 0.49% vs 3.70 ± 1.01%)	↓ *Lactococcus* (light phase) (2.66 ± 0.84% vs 0.45 ± 0.16%) ↓ *Lactobacillus* (dark phase) (3.62 ± 1.49% vs 0.06 ± 0.04%)		↔	√
Zarrinpar et al, 2014^10^	Cecum sample; every 4 h over 24 h (ZT1, 5, 9, 13, 17, 21)	HFD TRE vs CD AL	↔Firmicutes ↔Bacteroidetes ↔Veruccomicrobia	NR		↘ *Lactobacillus* (0.97 ± 0.49% vs 3.70 ± 1.01%)	NR		↓	√
Ye et al, 2020^11^	Rectal samples; ZT0, ZT8, ZT12, and ZT20	HFD TRE vs HFD AL	↘ Firmicutes (58.04 ± 9.33% vs 34.10 ± 13.49%) ↗ Bacteroidetes (39.28 ± 17.08% vs 27.02 ± 13.06%) ↔ Proteobacteria *↔* Actinobacteria	Light phaseZT0ZT8	↔ Firmicutes ↔ Bacteroidetes ↔ Firmicutes ↔ Bacteroidetes	NR	NR			
				Dark phaseZT12ZT20	↔ Firmicutes ↔ Bacteroidetes ↓ Firmicutes (35.04 ± 9.38% vs 52.77 ± 7.73%) ↑Bacteroidetes (57.58 ± 10.77% vs 29.27 ± 11.56%)					
Ye et al, 2020^11^	Rectal samples; ZT0, ZT8, ZT12, and ZT20	HFD TRE vs CD AL	↗ Firmicutes (47.89 ± 12.86% vs 34.1 ± 13.49%) ↘ Bacteroidetes (39.28 ± 17.08% vs 61.34 ± 12.99%) ↗ Proteobacteria (9.471 ± 5.918% vs 2.34 ± 1.38%) ↔ Actinobacteria	Light phaseZT0ZT8	↑ Firmicutes ↓ Bacteroidetes ↔ Firmicutes ↔ Bacteroidetes	NR	NR		↔	√
				Dark phaseZT12ZT20	↑ Firmicutes ↓ Bacteroidetes ↔ Bacteroidetes ↔ Firmicutes					
He et al, 2021^14^	Cecal samples after 4 wk of LD	LD RF-like vs LD AL	NR	Light phaseZT0ZT4ZT8	↑ Firmicutes ↓ Veruccomicrobia ↔ Actinobacteria ↔ Proteobacteria ↔ Bacteroidetes ↑ Proteobacteria ↔ Firmicutes ↔ Veruccomicrobia ↔ Actinobacteria ↔ Bacteroidetes ↑ Firmicutes ↓Bacteroidetes ↑ Proteobacteria ↑ Actinobacteria ↔ Veruccomicrobia	NR	NR		NR	√
				Dark phaseZT12ZT16ZT20	↑ Proteobacteria ↓ Actinobacteria ↔ Veruccomicrobia ↔ Firmicutes ↔ Bacteroidetes ↑ Bacteroidetes ↑ Actinobacteria ↔ Veruccomicrobia ↔ Firmicutes ↔ Proteobacteria ↔ Veruccomicrobia ↔ Firmicutes ↔ Actinobacteria ↔ Proteobacteria ↔ Bacteroidetes					
Machado et al, 2022^24^	Ileal samples; ZT1, ZT4, ZT9, ZT13, ZT17, ZT21; after HFD	HFD TRE vs HFD AL	↔ Bacteroidetes			*↑ Enterococcus* *↑ Staphylococcus* *↑ Lactococcus* *↑ Colidextribacter* *↑ Blautia*	Light phase	*↑ Ruminococcaceae/Lactococcus* *↓ Turicibacter/Enterococcus* *↑Enterococcus/Lactococcus*	↔ (Shannon index) ↔ (Faith’s PD)	√
						*↑ Tuzzerella* *↑ Angelakisella* *↑ Helicobacter* *↑ Parasutterella* *↓ Ruminococcus* *↓ Lachnoclostridium* *↓ Turicibacter* *↓ Alistipes* *↓ Akkermansia*	Dark phase	*↔ Ruminococcaceae/Lactococcus* *↓ Turicibacter/Enterococcus* *↔ Enterococcus/Lactococcus* ZT13 *↑ Staphylococcus*		
Machado et al, 2022^24^	Ileal samples; ZT1, ZT4, ZT9, ZT13, ZT17, ZT21; after HFD	HFD TRE vs CD AL	↘ Bacteroidetes			*↑ Staphylococcus* *↑ Leuconostoc* *↑ Colidextribacter* *↑ Blautia* *↑ Enterococcus* *↑ Tuzzerella* *↑ Lactococcus* *↓ Turicibacter* *↓ Lachnoclostridium* *↓ Ruminococcus* *↓ Monoglobus* *↓ Alistipes*	Light phase	*↓ Ruminococcaceae/Lactococcus* *↓ Turicibacter/Enterococcus* *↔ Enterococcus/Lactococcus*	↔ (Shannon index) ↓ (Faith’s PD)	√
							Dark phase	*↓ Ruminococcaceae/Lactococcus* *↓ Turicibacter/Enterococcus* *↓ Enterococcus/Lactococcus* ZT13 *↔ Staphylococcus*		
Machado et al, 2022^24^	Cecal samples; ZT1, ZT4, ZT9, ZT13, ZT17, ZT21; after HFD	HFD TRE vs HFD AL	NR	NR		NR	NR		↔ (Shannon index) ↔ (Faith’s PD)	√
Machado et al, 2022^24^	Cecal samples; ZT1, ZT4, ZT9, ZT13, ZT17, ZT21; after HFD	HFD TRE vs CD AL	NR	NR		NR	NR		↔ (Shannon index) ↓ (Faith’s PD)	√
Termination at 1 ZT point										
Hu et al, 2018^14^	Cecal samples; ZT21; after CD	CD TRE vs CD AL	↗ Firmicutes ↘ Bacteroidetes			↗ *Lactobacillus* *↗ Roseburia* ↘ *Staphylococcus*			↔	√
Li et al, 2020^27^ (16 h of fasting)	Fecal samples; day 30 and day 60; after CD	CD TRE vs CD AL	NR			↗ *Akkermansia* ↘ *Alistipes*			↔	√
Li et al, 2020^27^ (12 h of fasting)	Fecal samples; day 30 and day 60; after CD	CD TRE vs CD AL	No taxonomic differences			No taxonomic differences			↔	√
Li et al, 2020^27^ (20 h of fasting)	Fecal samples; day 30 and day 60; after CD	CD TRE vs CD AL	No taxonomic differences			No taxonomic differences			↔	√
van der Merwe et al, 2020^16^	Fecal samples; after 6 wk of HFD (T0) and again at 3 wk (T1) & 7 wk (T2)	HFD TRE vs HFD AL	NR			↗ *Ruminococcus* ↗ *Lactococcus* ↗ *Desulfovibrio* ↗ *Enterococcus*			↑	x
	Cecal samples; after HFD	HFD TRE vs HFD AL	↗ Verrucomicrobia (6%) (unknown significance)			↗ *Lactococcus* ↗ *Akkermansia* ↘ *Bilophila*			NR	x
Palomba,2021^26^	Fecal samples; after 48 wk of CD	CD TRE vs CD AL	NR			↗ *Akkermansia* (number of readings - 4327 vs 15) ↘ *Bilophila* (52 vs 21) ↘ *Lactococcus* (386 vs 46) ↘ *Ruminococcus* (11 948 vs 2979) ↘ *Turicibacter* (576 vs 167) ↔ *Lactobacillus* *↔ Oscillibacter* *↔ Roseburia* *↔ Alistipes* *↔ Desulfovibrio* *↔ Colidextribacter* *↔ Blautia* *↔ Tuzzerella* *↔ Angelakisella* *↔ Helicobacter* *↔ Parasutterella* *↔ Lachnoclostridium* *↔ Monoglobus*			NR	NR
Su et al, 2022^28^	Fecal samples; day 0 and day 30; after CD	CD RF-like vs CD AL	↗ *Firmicutes* (52.79 ± 7.48% vs 67.53 ± 4.84%) ↘ Bacteroidetes (38.79 ± 4.93% vs 24.39 ± 6.12%)			NR			↔	√

*Abbreviations*: AL, ad libitum; CD, chow (feed-pellet) diet; HFD, high-fat diet; LD, lithogenic diet containing 1.25% cholesterol and 0.5% cholic acid; NR, not reported; PD, phylogenetic diversity; RF-like, Ramadan-like fasting; TRE, time-restricted eating; ZT, zeitgeber time; ↓, significant decrease; ↔, nonsignificant effect; ↑, significant increase; ↗, enrichment in comparison to control group; ↘, depletion in comparison to control group; √, changes in beta-diversity, X, no changes in beta-diversity.

**Table 5:** Effects of the TRE and RF regimens at the phylum and genus levels, and on the alpha- and beta-diversity, in human studies

Source	Phylum	Genus	Alpha-diversity	Beta-diversity
Time-restricted eating				
Gabel et al, 2020^25^	↔ Firmicutes ↔ Bacteroidetes	NR	↔	NR
Zeb et al, 2020^22^	Most abundant in the TRE group: Bacteroidetes, Firmicutes, Proteobacteria, Actinobacteria	Most abundant in TRE group: *Faecalibacterium*, *Dialister*, *Prevotella_9* Less abundant in TRE group: *Alloprevotella*, *Prevotella_7*, *Prevotella_2*	↑	NR
Zeb et al, 2020^21^	Most abundant in TRE group: Bacteroidetes	Most abundant in TRE group: *Prevotella_9*, *Prevotella_2*	↑	√
Xie et al, 2022^23^ (eTRE group)	No taxonomic differences	No taxonomic differences	↑	NR
Xie et al, 2022^23^ (mTRE group)	No taxonomic differences	No taxonomic differences	↔	NR
Ramadan fasting				
Ozkul et al, 2020^17^	*↑* Bacteroidetes	*↑ Roseburia* *↑ Akkermansia* *↑ Bacteroides* *↑ Butyricicoccus* *↑ Faecalibacterium*, *↑ Allobaculum*, *↑ Eubacterium*, *↑ Dialister*, *↑ Erysipelotrichi*	↑	√
Su et al, 2021^18^ (young cohort)	*↑* Firmicutes (40.56 ± 13.90% → 56.41 ± 14.49%) ↑ Proteobacteria (5.55 ± 4.56% → 7.85 ± 4.53%) ↓ Bacteroidetes (53.14 ± 15.81% → 34.37 ± 12.51%)	*↑ Faecalibacterium* (5.62 ± 2.96% → 9.47 ± 5.93%) ↓ *Prevotella_9* (21.79 ± 17.69% → 7.63 ± 10.20%)	↑	√
Su et al, 2021^18^ (middle-aged cohort)	NR	↗ *Agathobacter* (5.42 ± 4.56% → 12.57 ± 13.75%) ↗ *Blautia* (6.19 ± 6.36% → 9.71 ± 5.66%) ↘ *Megamonas* (1.53 ± 3.45% →0.04 ± 0.86%)	↔	√
Ali et al, 2021^29^	*↑* Proteobacteria	*↑ Klebsiella* *↓Coprococcus* *↓Clostridium_XIVa*	↔	X

*Abbreviations*: eTRE, early TRE; mTRE, mid-day TRE; NR, not reported; RF, Ramadan fasting; TRE, time-restricted eating; ↓, significant decrease; ↔, nonsignificant effect; ↑, significant increase; ↗, enrichment in comparison to control group; ↘, depletion in comparison to control group; √, changes in beta-diversity; X, no changes in beta-diversity.

### Effects of TRE and Ramadan-like fasting on composition, alpha-diversity, and beta-diversity of microbiota in the animal studies

Seven out of the 9 studies analyzed alpha-diversity using the Shannon,^10^^,^^11^^,^^14^^,^^16^^,^^24^^,^^28^ Chao1, ACE (abundance-based coverage estimator),^11^^,^^14^ Simpson,^14^^,^^27^^,^^28^ and Faith’s phylogenetic diversity (PD)^24^ indicators. Three studies that administered a feed-pellet diet under the TRE regimen found no changes in alpha-diversity.^14^^,^^27^ In the remaining 4 studies,^10^^,^^11^^,^^16^^,^^24^ where an HFD is administered following TRE, the alpha-diversity results were inconclusive. More specifically, in 2 studies,^10^^,^^11^ the TRE regimen was found to not alter alpha-diversity in animals consuming an HFD. The study by van der Merwe et al,^16^ on the other hand, was the only one in which the TRE regimen, despite an HFD, preserves microbiota diversity. However, in relation to the control groups fed the feed-pellet diet, Zarrinpar et al^10^ noted a decrease in alpha-diversity, while Ye et al^11^ found an increase in this parameter. Interestingly, the study by Machado et al^24^ found the result to be dependent on the alpha-diversity indicator used. No changes in the ileal or cecal contents were observed with the Shannon index, while Faith’s PD pointed to a decrease in alpha-diversity in the animals fed an HFD under the TRE regimen, as compared with the control animals fed a feed-pellet diet AL. Only 1 of the Ramadan-like fasting studies assessed the alpha-diversity, and found no changes.^28^

Cyclicality and fluctuations in the microbiota were also analyzed, but only in studies in which an HFD or lithogenic diet was administered (TRE^10^^,^^11^^,^^26^ or Ramadan-like fasting,^15^ respectively). For each operational taxonomic unit (OTU), the percentage of total reads was calculated for each mouse and then averaged per time point per condition. These data were analyzed to detect cyclical variation.^30^ Zarrinpar et al^10^ and Machado et al^24^ showed that the microbiome of mice fed an HFD under the TRE regimen exhibited greater fluctuations than the microbiome of the mice consuming an HFD given AL, despite using different approaches based on OTUs and amplicon sequence variants (ASVs), respectively. However, Zarrinpar et al^10^ noted that the number of OTUs that change cyclically was the same in both groups fed the HFD, whether by TRE or AL, and was lower than in the group fed the feed-pellet diet AL, while Machado et al observed that the number of cyclic ASVs in the HFD TRE group was similar to that in the feed-pellet diet AL group, and higher than in the HFD AL group. He et al^15^ noted a significant decrease in the variability of OTUs under the Ramadan-like fasting regimen, unlike Zarrinpar et al^10^ who use standard TRE. OTUs are defined as a cluster of sequences that have a sequence identity above a certain threshold, typically above 97%. On the other hand, ASV is an exact sequence variant or amplicon sequence variant, which is created as a result of a methodological change involving the increased use of denoising methods. Therefore, ASV-based approaches have a higher sensitivity in detecting bacterial strains present compared with OTUs, but sometimes at the expense of specificity.^31^

Beta-diversity was analyzed in 8 out of 9 studies using principal coordinate analysis based on Bray–Curtis distances,^16^^,^^24^^,^^27^^,^^28^ weighted UniFrac,^14^^,^^15^^,^^24^ or the Jaccard dissimilarity index.^10^ One study uses principal component analysis.^11^ Seven studies in which animals were fed an HFD/feed-pellet diet^10^^,^^11^^,^^14^^,^^24^^,^^27^ with TRE or a lithogenic/feed-pellet diet under a Ramadan-like fasting regimen^15^^,^^28^ showed differences in beta-diversity between the intervention (feed deprivation) and control (AL) groups. Interestingly, after feeding an HFD with TRE, Ye et al^11^ observed a difference only in relation to the feed-pellet diet given AL. In turn, van der Merwe et al^16^ noted no such changes between groups fed an HFD with TRE or AL.

All of the animal studies showed the relative abundances of the 2 taxonomic levels—phylum and genus, and all of the changes discussed were statistically significant unless otherwise noted. The data are presented in Table 4. The TRE studies in which animals were fed a feed-pellet diet showed a growth trend in the Firmicutes phylum. In fuller detail, 2 studies showed a greater abundance of the Firmicutes phylum in the TRE group fed a feed-pellet diet or HFD than in the case of the feed-pellet diet given AL.^11^^,^^14^ Interestingly, an increase in the Firmicutes phylum was noted in the Ramadan-like fasting studies of He et al^15^ and Su et al,^28^ in which the feeding window with the lithogenic or feed-pellet diet was during the light (resting) phase. The results for the Bacteroidetes phylum were the opposite in the TRE and RF-like studies.^11^^,^^14^^,^^15^^,^^24^^,^^28^ In 1 study in which animals were fed an HFD under a TRE regimen, the abundance of Firmicutes was notably lower—by approximately 23.9% in total and by approximately 17.7% at the ZT20 point (dark phase)—than in the animals fed the HFD AL.^11^

Interestingly, in the microbiota of the mice fed an HFD according to the TRE regimen, Ye et al^11^ and Zarrinpar et al^10^ observed an increase in the fluctuations of Firmicutes and Bacteroidetes, compared with both control groups (HFD and feed-pellet diet given AL, respectively).^10^^,^^11^ On the other hand, He et al^15^ noted a decrease in microbiota fluctuations, especially with regard to Bacteroidetes and Firmicutes, after a lithogenic diet during the resting phase (Ramadan-like fasting).

At the genus level, 1 study found a greater abundance of *Lactobacillus* (by 854 reads) in animals consuming a feed-pellet diet under the TRE regimen than in animals fed this diet al.^14^ Zarrinpar et al^10^ provided time-restricted access to the HFD diet and observed a decrease in the relative abundance of *Lactobacillus* (by 2.7%), in comparison to the control groups fed both a feed-pellet diet and an HFD given AL. Interestingly, Zarrinpar et al also found that TRE has a beneficial effect on maintaining the cyclic variability of this genus, as no changes were observed in the group consuming the feed-pellet diet, while an increase in cyclicity was observed in the group consuming the HFD. In turn, Machado et al^24^ observed that the cyclicity of *Lactobacillus* was preserved only in the groups fed the HFD diet (whether AL or TRE), but that in the TRE group there was a sharp decrease in fluctuations in *Lactobacillus* in the dark phase compared with the HFD AL group.

The results regarding the effects of TRE on *Lactococcus* were inconclusive. In the microbiota of mice fed an HFD in the TRE regime, Zarrinpar et al^10^ noted a decrease (by 2.2%) in the relative abundance of *Lactococcus* in comparison to the group fed an HFD AL. This contrasts with the findings of van der Merwe et al^16^ and Machado et al^24^ who observed an overall greater relative abundance of this genus in mice also fed an HFD under TRE. Interestingly, in 1 study by Zarrinpar et al,^10^ TRE with an HFD halted oscillations in the relative abundance of *Lactococcus*, while the study by Machado et al^24^ noted both *Lactococcus* and *Staphylococcus* oscillations only in the TRE HFD group. Furthermore, *Oscillibacter* oscillations were disturbed by an HFD, regardless of the dietary regimen,^10^ while *Streptococcus* oscillations in HFD TRE were the same as in the feed-pellet diet AL group and greater than for the HFD AL.^24^ Moreover, van der Merwe et al^16^ also showed a greater abundance of *Ruminococcus* in mice fed an HFD in TRE than in the control HFD AL group. In turn, in a study by Palomba,^26^ mice fed a feed-pellet diet under TRE were observed to have significantly lower abundances of the *Ruminococcus* genus than a group fed a feed-pellet diet given AL. Interestingly, although it can be assumed that the discrepancy in *Ruminococcus* might be due to differences in the type of diet used (HFD and feed-pellet diet), Machado et al^24^ also noted a decrease in the abundance of *Ruminococcus* in the TRE HFD group compared with both AL-fed groups (feed-pellet diet and HFD).

### Effects of TRE on the composition, alpha-diversity, and beta-diversity of microbiota in human studies

Two human studies observed significantly higher alpha-diversities^21^^,^^22^ in the TRE group, while, in 1 study, the alpha-diversity remained unchanged after introducing the dietary regimen.^25^ In the study by Xie et al,^23^ significantly higher alpha-diversity was observed only in the early TRE group and not in the nonfasting control; the mid-day TRE group showed no changes. To assess alpha-diversity, these studies used the Shannon index,^21^^,^^25^ the Richness index,^21^^,^^22^^,^^25^ and the Chao1 indicator.^23^

Beta-diversity was examined using principal component analysis and principal coordinate analysis with Bray–Curtis distance measurement in 1 study by Zeb et al,^21^ while the other study by the same authors used the UniFrac distance.^22^ Only the first of these studies^21^ showed differences between the TRE and control groups.

All of the human TRE studies presented the relative abundance of microbiota (Table 5) and all changes discussed here were statistically significant unless otherwise noted. In TRE studies at the phylum level, Bacteroidetes was the more abundant phylum, unlike in the control group.^21^^,^^22^ However, in the study conducted by Gabel et al,^25^ no changes were observed in this phylum after dietary intervention.

Analysis at the genus level was performed in both studies conducted by Zeb et al; in one of these, *Faecalibacterium* and *Dialister* were more abundant in the TRE group than in the nonfasting control group. Changes in the *Prevotella* genus were also observed. *Prevotella_9* was most abundant in both studies by Zeb et al,^21^^,^^22^ while the results for *Prevotella_2* were inconclusive: 1 study showed the most abundance,^21^ and another study showed less abundance, in the TRE group, compared with the nonfasting control group.^22^ Moreover, Xie et al^23^ did not observe any changes at either taxonomic level.

### Effects of RF on the composition, alpha-diversity, and beta-diversity of microbiota in human studies

Two human studies^17^^,^^18^ observed an increase in the alpha-diversity over the baseline parameters, while another study noted no changes with respect to either the baseline parameters^29^ or to the nonfasting control group (a middle-aged cohort).^18^ To assess the alpha-diversity, these studies used the Shannon index,^17^^,^^18^^,^^29^ Simpson’s index,^18^^,^^29^ OTUs Richness,^17^ the Chao1 index, and the ACE index.^29^

Beta-diversity was analyzed in all of the studies using principal coordinate analysis, based either on Bray–Curtis distances^18^^,^^29^ or on the unweighted and weighted UniFrac algorithm^17^; nonetheless, significant differences were only seen in 2 studies.^17^^,^^18^

All of the human RF studies presented relative abundances on the 2 taxonomic levels—phylum and genus—and all changes discussed here were statistically significant unless otherwise noted. The data are presented in Table 5. In 1 of the 3 studies, an increase of 18.8% in Firmicutes at the phylum level was observed in the younger cohort over the pre-RF value.^18^ Furthermore, both of the studies by Ali et al^29^ and Su et al,^18^ which were conducted among young cohorts, observed increases in Proteobacteria, by 4.7% and 2.3%, respectively. One study by Ozkul et al^17^ reported an increase in Bacteroidetes, while another by Su et al^18^ described a decrease in this phylum by 18.8% compared with baseline values. Moreover, the study by Ozkul et al^17^ was the only one to assesses the Firmicutes:Bacteroidetes (F/B) ratio; this proved to be elevated both before and after the application of the Ramadan dietary habits, so no changes were observed.

One the genus level, an increase in *Faecalibacterium* (by 3.9%) was observed in 2 of 3 studies.^17^^,^^18^ There was also an increase in the relative abundance of *Roseburia*, *Akkermansia*, *Bacteroides*, *Butyricicoccus*, *Allobaculum*, *Eubacterium*, *Dialister*, *Erysipelotrichi*,^17^ and *Agathobacter* by 7.2%; of *Blautia* by 3.5%^18^; and of *Klebsiella*^29^ compared with baseline. In turn, a decrease in *Prevotella_9* by 14.2% and in *Megamonas* by 1.5% was also observed by Su et al.^18^

### Associations between composition of the microbiota and host metabolic markers caused by TRE or RF in human studies

Only 2 studies^18^^,^^21^ showed any correlation between host metabolic markers or body weight and gut microbiota composition. In one of the studies conducted by Zeb et al,^21^ a positive relationship was shown between HDL concentration and the richness of the intestinal microbiome after TRE (*r* = 0.42, *P* = .0289). Su et al^18^ further observed a positive correlation between body mass index values and the abundance of OTUs belonging to the phylum Proteobacteria alongside a negative correlation between body mass index and abundance of the class Negativicutes and the order Selenomonadales after RF (*P* < .05).

## DISCUSSION

It is accepted that the feeding–fasting cycle affects host metabolism^10^; however, little is known regarding the essential characteristics of the changes that occur in the gut microbiota, and even less about the correlation between microbiota changes and host metabolic parameters. This study is the first systematic review to summarize the effects of TRE and RF on specific taxonomic groups of gut microbiota and to examine the correlations between the composition of microbiota and host metabolic parameters in both humans and animals.

This systematic review reveals that TRE may restore the cyclical fluctuation of major phyla within the gut microbiome of mice fed an HFD.^10^^,^^11^ However, TRE in the presence of an HFD does not lead to the microbial dynamic becoming as dynamic as is observed in mice fed a feed-pellet diet,^10^^,^^11^ indicating that diet is an important factor in forming the gut microbial environment. Ye et al^11^ indicated that the circadian microbial rhythm of mice fed an HFD given under the TRE regimen is opposite to mice fed an HFD AL. At the same time, the major microbial phyla in the mice fed the feed-pellet diet AL oscillate with a diurnal pattern: During the night, when rodents are active, the Firmicutes count is at its highest, while Bacteroidetes are lower in number; during the day, when the rodents are resting, the latter have higher numbers.^11^ These rhythmic changes in the abundance of major phyla may occur on account of the fact that Firmicutes are more effective than Bacteroidetes at obtaining energy from food (hence, they increase when food is consumed). This would explain why both the abundance of bacteria of the Firmicutes phylum and greater F/B ratio are associated with obesity.^32^ It should be noted that the introduction of an eating window during the resting phase (Ramadan-like fasting) in mice also results in an inversion of the circadian rhythm of these major phyla.^15^

Cyclical fluctuations in specific members of the gut microbiota contribute to microbial diversity, and likely represent a mechanism by which the microbes affect the host’s metabolism.^10^ Indeed, alpha-diversity is the most common means of assessing not only intestinal microbiota health but also human nutritional status,^33^ with lower levels of diversity being associated with obesity and metabolic syndrome.^34^ Moreover, it is well known that dietary fiber intake has direct effects on the amount of microbial diversity in the gut.^35^ There is no difference in animal studies in alpha-diversity between animals fed AL or those on TRE, regardless of the diet administered. This may be due to the composition of both the intervention and control diets, as Wang et al^8^ noted that the increase in alpha-diversity in HFD-fed mice was due to a greater amount of fiber in this diet than in the control diet. Interestingly, in the human studies, the use of both TRE and RF was related to an increase in gut microbial community diversity. Meals eaten during Ramadan contain more foods rich in carbohydrate and fiber,^36^ such as soups, porridges, legumes, and whole grains,^37^ which could partially explain the difference in gut microbial diversity before and after RF. Unfortunately, dietary fiber consumption was not assessed in any of the RF studies examined here, while in TRE studies, Zeb et al^21^^,^^22^ did not show any difference in fiber intake. The lack of change is probably due to the fact that TRE does not impose any restrictions on diet quality^11^; however, animal studies where the quality of the diet is under control allow us to examine the relationship between TRE and the quality of the diet.

In turn, changes in beta-diversity were observed mainly in animal studies using TRE, as well as in the 2 of 3 human studies that tested RF.^17^^,^^18^ While alpha-diversity as a measure of microbiome diversity is applicable to a single sample, beta-diversity is a measure of the similarity or dissimilarity of 2 communities.^38^ It can thus be pointed out that fluctuations in the gut microbiome are important for host metabolism, and not necessarily for species richness, which is determined mainly by diet.^10^

This systematic review shows that TRE alters the average abundance of the main microbial phyla—Firmicutes and Bacteroidetes. In animal studies, the direction of such changes may be associated with the kind of diet that is given under the TRE regimen (HFD^10^^,^^11^^,^^24^ or feed-pellet diet^14^^,^^28^). In 2 of the 4 human TRE studies, Bacteroidetes were more abundant than in the nonfasting control groups,^21^^,^^22^ while the human RF studies were inconsistent.^17^^,^^18^ Bacteria of the Bacteroidetes phylum are responsible for the production of acetic acid, which can be successfully converted to butyric acid—although only if the microbiome has the appropriate F/B ratio.^39^ Furthermore, acetic acid accumulates in the hypothalamus and, through a series of reactions, suppresses appetite, which may be useful in the treatment of obesity.^39^ Moreover, obesity is associated with an elevated F/B ratio.^40^ There is also a correlation between this indicator and eating behaviors^41^; it can thus be suggested that, in the case of people with obesity who often snack under the influence of emotions,^42^ regardless of the time of day or feelings of hunger,^43^ the optimal F/B ratio may be disturbed by the virtually uninterrupted availability of foods.^44^ The human study by Ozkul et al^17^ was the only one to evaluate the F/B ratio and note that it remained high after RF, so no significant changes were observed (data not shown).

In 2 studies in which animals were fed the HFD or lithogenic diet under the TRE^11^ and RF-like regimens,^11^^,^^15^ an enrichment in the Proteobacteria phylum was seen. Similar changes were observed in the human RF studies.^15^^,^^25^ The effect of Proteobacteria on the body’s functioning seems to be controversial: on one hand, it has been suggested that Proteobacteria contribute to homeostasis of the anaerobic environment in the gut tract, and thus to the stability of the strictly anaerobic microbiota^45^; on the other hand, an increase in abundance of Proteobacteria may be associated with metabolic syndrome.^46^

At the genus level, *Akkermansia* abundance seems to be dependent on the type of diet administered in TRE (enrichment after a feed-pellet diet^16^^,^^26^^,^^27^ and decrease after an HFD^24^). On the other hand, only the RF study by Ozkul et al^17^ among the human studies showed an increase in the *Akkermansia* genus. An increase in the abundance of these bacteria seems to be extremely favorable, as it has been noted that *Akkermansia muciniphila* causes an increase in the expression of genes associated with immune responses and in the strengthening of the gut barrier function.^44^ It is also indicated that *A. muciniphila* affects glucose and lipid metabolism through the production of mucin, which improves the strength of the intestinal barrier and stimulates the immune system to secrete anti-inflammatory cytokines.^47^ Moreover, although *A. muciniphila* is a G(-) bacterium, it is not associated with endotoxemia and, more importantly, it reduces the concentration of endotoxins resulting from consuming an HFD.^46^*Akkermansia muciniphila* is also inversely correlated with the occurrence of insulin resistance and obesity.^47^

The increase in the abundance of Faecalibacterium, which was observed only in human RF studies, and their significantly greater abundance than in the control group, is also interesting.^17^^,^^18^^,^^22^ By producing butyric acid and other short-chain fatty acids, this genus is strongly associated with intestinal health and also has a strong anti-inflammatory effect. Less abundant Faecalibacterium is observed in individuals with irritable bowel syndrome^48^ and in those with depression^49^ or Parkinson’s disease.^50^ It can therefore be suggested that this change caused by RF seems to be beneficial.

The results for the *Lactobacillus* genus are inconclusive. In the animal studies alone, consumption of an HFD under TRE was associated with a decrease in the abundance of this genus,^10^ while the intake of a control diet led to either a greater abundance of this genus^14^ or no change.^26^ In general, *Lactobacillus* is associated with good intestinal health,^51^ because it strengthens the intestinal barrier function by increasing mucus production or stimulating release of antimicrobial peptides and providing a competitive resistance against pathogens.^52^ However, further studies are needed to determine whether these changes are directly related to the dietary regimen or just to the type of diet.

There are some reports that suggest that beneficial changes in host metabolic parameters may be the direct result of changes in the microbiota induced by TRE. For instance, Wang et al^53^ showed that the TRE-dependent increase in *Prevotellaceae* abundance in the microbiome of swine was negatively correlated with blood levels of 2-amino-butyrate, suggesting a reduced risk of cardiovascular disease. Zeb et al^21^ suggested that TRE reduces the risk of developing metabolic disease precisely by regulating the level of serum HDL caused by the microbiome in humans, while Su et al^18^ indicated that RF can be associated with beneficial changes in body mass index. Unfortunately, the small number of studies associating microbiota changes with improvements in metabolic or anthropometric parameters induced by TRE makes it impossible to unequivocally state whether the observed microbial and metabolic changes are actually related.

The articles included in this systematic review have some limitations. In the human studies that examined fecal microbiota composition, it can be difficult to determine the exact time and method of collection (eg, sample storage and process sterility); in many cases, this leads to an inability to compare results between studies.^54^ These differences undoubtedly have a large effect on the measured microbiome composition. The same consideration applies to the examination of animal feces, as it is also not possible to collect them immediately after expulsion. Furthermore, assessing human or animal gut microbiota composition at 1 point in time (whether fecal or intestinal) makes it impossible to observe cyclical circadian fluctuations in the microbiota. The results of the included studies lead us to conclude that the microbiota can be most accurately assessed from the intestinal contents collected at circadian termination.

Some studies show that different primer pairs may affect the microbial profile. Primers spanning more than 1 V region generally enhance precision in identifying bacteria, as compared with a single region. The studies reviewed in this article mostly used the V3-V4 or V1-V3 regions. It has been shown that the V3-V4 region slightly outperforms the other region combinations, and thus might be recommended for the analysis of human gut samples.^55^ Another limitation of this review is that the studies it considers are based not only on different study populations (humans and animals) but also on different intervention protocols with, for example, eating windows being during the day or during night; this may cause some ambiguity and make interpretation difficult.

Moreover, although only 2 human studies were assessed negatively as “fair”^21^^,^^22^ and the rest of the studies were assessed as “good,” there are some aspects that, although not covered by the tools used, could improve the quality of the research. First, no animal study evaluated fiber intake. Although 5^17^^,^^18^^,^^21^^,^^22^^,^^29^ of the 7 human studies assessed the composition of the diet, 3 of them also did not assess fiber consumption.^17^^,^^18^^,^^29^ The precise estimation of fiber consumption in both human and animal studies is indeed a valuable result, but the opportunity to detect a correlation between the consumption of individual nutrients and the composition of microbiota should not be overlooked, as it would increase the quality of these studies.

This study also has a number of strengths. To the best of knowledge, ours is the first study to discuss the changes in microbiota composition caused by TRE and RF in both animal and human studies. This systematic review includes both preclinical studies in animals and preliminary studies in humans, in order to discuss the effects and potential differences resulting not only from genetic variation but also from the material collected for the microbiome study.

## CONCLUSION

These findings support the importance of TRE and RF in improving gut microbiota composition. However, based on the results of animal studies, it can be suggested that diet remains the essential factor in forming its environment. Since only a small number of studies link changes in the microbiota with improvements in metabolic or anthropometric parameters induced by the regimens studied, it is impossible to unequivocally state whether all the observed microbial and metabolic changes are actually related. Further research should thus include metagenomics and microbial and host metabolomics in their methodology to better understand the potential correlations between microbes and host health. It should be pointed out that data in this field remain limited, especially among human studies, and so it is difficult to draw meaningful conclusions about the effects of the TRE and RF on specific taxonomic groups of gut microbiota. Moreover, more precise inspection of the human diet and of the time of specimen collection is necessary to better interpret studies of the gut microbiome, and to better understand the host–microbiome relationship.

## Supplementary Material

nuad093_Supplementary_Data

## Supporting Information

The following Supporting Information is available through the online version of this article at the publisher’s website.

*Table S1* **Evaluation of the quality of the animal studies**

*Table S2* **Evaluation of the quality of the human studies**

*Table S3* **Effects of time-restricted eating (TRE) regimen at the phylum and genus level, and in alpha and beta diversity, in animal studies**

*Table S4* **PRISMA checklist**
