# Supplementary material for: The effects of time-restricted eating and Ramadan fasting on gut microbiota composition: a systematic review of human and animal studies
Source: Nutr Rev. 2023 Aug 1;82(6):777–93. doi: 10.1093/nutrit/nuad093 (PMC11082590; doi:10.1093/nutrit/nuad093)
Supplement: nuad093_Supplementary_Data [file nuad093_supplementary_data.zip › nuad093_Supplementary_Data/26.06 PRISMA Checklist.docx]

# PRISMA 2020 Main Checklist

| **Topic** | **No.** | Item | Location where item is reported |
| --- | --- | --- | --- |
| **TITLE** |  |  |  |
| **Title** | 1 | Identify the report as a systematic review. | The effects of time-restricted eating and Ramadan fasting on gut microbiota composition: A systematic review of human and animal studies |
| **ABSTRACT** |  |  |  |
| **Abstract** | 2 | See the PRISMA 2020 for Abstracts checklist | *Context:*It is well known that the microbiome undergoes cyclical diurnal rhythms. It has thus been hypothesized that meal timing may affect gut microbial composition, function, and host health.*Objective*:This review aim to examine the effects of time-restricted eating (TRE) and Ramadan fasting (RF) on the composition of the gut microbiota in animal and human studies. The associations between composition of microbiota and host metabolic parameters are also examined. *Data Sources*:A search was performed on the PubMed, Cochrane, Scopus, and Web of Science databases up to December 31, 2022. The search strategy was performed using the Medical Subject Heading (MeSH) terms “intermittent fasting” and “gastrointestinal microbiome” and the keywords “ramadan fasting” and “microbes”.*Data Extraction*:Seven human studies (four TRE and three RF) and nine animal studies (seven TRE, two Ramadan-like fasting) were retrieved. *Data Analysis*: TRE and RF in human studies lead to an increase in gut microbial community alpha-diversity. In the animal studies (both TRE and RF-like), fasting is not associated with improved alpha-diversity, but enhancement of microbial fluctuation is observed, compared to high-fat diet *ad libitum* groups. Within *Firmicutes* and *Bacteroidetes* phyla, no specific direction of changes resulting from fasting are observed in both animals and human. After TRE or RF, a greater abundance of the *Faecalibacterium* genus is observed in human studies; changes in *Lactobacillus* abundance are found in animal studies; and increases in *Akkermansia* are seen both in humans and in animals fed a chow diet. Only two human studies show a beneficial correlation between microbiota changes and host metabolic (HDL-C) or anthropometric parameters (BMI).*Conclusions*:These findings support the importance of both regimens in improving the gut microbiota composition. However, based on animal studies` results, it can be suggested that diet remains the essential factor in forming the microbiota’s environment. *Systematic Review Registration:* PROSPERO registration number CRD42021278918 |
| **INTRODUCTION** |  |  |  |
| **Rationale** | 3 | Describe the rationale for the review in the context of existing knowledge. | Circadian rhythms represent an endogenous time-keeping system that regulates and synchronizes behavior, physiology, and metabolism with external cues known as zeitgebers (ZT), to establish homeostasis. The light/dark cycle is the most important zeitgeber, but other stimuli such as temperature and the presence of food can also act as zeitgebers. It is well known that the microbiome undergoes cyclical diurnal rhythms. It has thus been hypothesized that meal timing may affect the gut microbial composition, function, and health of the host. One dietary regimen that can predispose to entraining peripheral oscillations is time-restricted eating (TRE), a feeding pattern where food intake is restricted to certain hours of the day (most often an eight-hour period), with no limitation on nutrient quality or quantity. One form of TRE is Ramadan feeding (RF), a regimen that is common among Muslims. |
| **Objectives** | 4 | Provide an explicit statement of the objective(s) or question(s) the review addresses. | This review aim to examine the effects of time-restricted eating (TRE) and Ramadan fasting (RF) on the composition of the gut microbiota in both animal and human studies. The associations between composition of microbiota and metabolic parameters are also examined. |
| **METHODS** |  |  |  |
| **Eligibility criteria** | 5 | Specify the inclusion and exclusion criteria for the review and how studies are grouped for the syntheses. | Any interventional and observational studies that met the following eligibility criteria are included: 1) The study participants are humans aged 18–65 years or rodents older than six weeks who underwent TRE or RF for at least three weeks; outcomes included changes in the composition of gut microbiota at different taxonomic levels (assessed by 16S rRNA) and its alpha diversity. Selected associations between the composition of the gut microbiota and metabolic parameters or body weight (secondary outcomes) were also evaluated. Systematic reviews, case reports, articles written in a language other than English, and papers in which a treatment arm (other than TRE/RF) included exercise, calorie restriction, or weight loss supplementation are excluded. The studies are grouped into animal and human studies. |
| **Information sources** | 6 | Specify all databases, registers, websites, organisations, reference lists and other sources searched or consulted to identify studies. Specify the date when each source is last searched or consulted. | A search is performed by JP-Z and JB on the PubMed, Cochrane, Scopus, and Web of Science databases up to December 31, 2022. The search strategy is performed using both Medical Subject Heading (MeSH) terms and keywords: The search for time-restricted eating employed the terms “intermittent fasting” (a MeSH term) OR “ramadan fasting” (a keyword). For gut microbiota, the search is carried out using the terms “gastrointestinal microbiome” (a MeSH term) OR “microbes” (a keyword). |
| **Search strategy** | 7 | Present the full search strategies for all databases, registers and websites, including any filters and limits used. | The search for time-restricted eating employed the terms “intermittent fasting” (a MeSH term) OR “ramadan fasting” (a keyword). For gut microbiota, the search is carried out using the terms “gastrointestinal microbiome” (a MeSH term) OR “microbes” (a keyword). |
| **Selection process** | 8 | Specify the methods used to decide whether a study met the inclusion criteria of the review, including how many reviewers screened each record and each report retrieved, whether they worked independently, and if applicable, details of automation tools used in the process. | The search results from all the databases are collected in the Mendeley tool, where duplicates are removed. A two-phase search strategy is subsequently employed by two independent reviewers (JP-Z and JB) up to December 31, 2022. In phase one, the eligibility of each study is assessed on the basis of its title and abstract. Studies that had questionable suitability are provisionally included, with a final decision made in phase 2. In phase 2, full articles are retrieved and assessed against the eligibility criteria. Reference lists of original and review articles are screened to ensure that all relevant studies had been included. Any disagreement over the eligibility of an article for this study is resolved through discussion with KŁ, AM and NL. |
| **Data collection process** | 9 | Specify the methods used to collect data from reports, including how many reviewers collected data from each report, whether they worked independently, any processes for obtaining or confirming data from study investigators, and if applicable, details of automation tools used in the process. | The search results from all the databases are collected in the Mendeley tool, where duplicates are removed. A two-phase search strategy is subsequently employed by two independent reviewers (JP-Z and JB) up to December 31, 2022. In phase one, the eligibility of each study is assessed on the basis of its title and abstract. Studies that had questionable suitability are provisionally included, with a final decision made in phase 2. In phase 2, full articles are retrieved and assessed against the eligibility criteria. Reference lists of original and review articles are screened to ensure that all relevant studies had been included. Any disagreement over the eligibility of an article for this study is resolved through discussion with KŁ, AM and NL. |
| **Data items** | 10a | List and define all outcomes for which data are sought. Specify whether all results that are compatible with each outcome domain in each study are sought (e.g. for all measures, time points, analyses), and if not, the methods used to decide which results to collect. | The following data are extracted from the animal studies: author, type of animal model, number of animals and their age, type of intervention, control conditions, intervention diet and duration of the study. The following outcomes are extracted from the animal studies: the type of material (colonic or fecal) taken to test the composition of the microbiota, the variable gene region selected for gene sequencing, the abundance of microbial taxa at the phylum and genus level, the alpha-diversity and beta-diversity parameters, as well as other study findings, such as associations between changes in microbiota and metabolic markers.  The following data are extracted from the human studies: author, study design, number of participants, age (years), type of intervention, control conditions, and duration of the study. The following outcomes are extracted from the human studies: the type of material (feces), variable gene region selected for gene sequencing, the abundance of microbial taxa at phylum and genus level, alpha-diversity and beta-diversity parameters, and other study findings, such as associations between changes in microbiota and metabolic markers.  Any disputes regarding the appropriateness of including or excluding a given study are resolved by discussion between the authors. |
|  | 10b | List and define all other variables for which data are sought (e.g. participant and intervention characteristics, funding sources). Describe any assumptions made about any missing or unclear information. | None |
| **Study risk of bias assessment** | 11 | Specify the methods used to assess risk of bias in the included studies, including details of the tool(s) used, how many reviewers assessed each study and whether they worked independently, and if applicable, details of automation tools used in the process. | For the rodent model studies, the Systematic Review Centre for Laboratory animal Experimentation (SYRCLE) risk-of-bias assessment tool is used. 18 For the human studies the Quality Assessment Tool for Observational Cohort and Cross-Sectional Studies, the Quality Assessment Tool for Before-After (Pre-Post) Studies With No Control Group, and the Quality Assessment of Controlled Intervention Studies from NIH National Heart, Lung, and Blood Institute 19 were used. Two independent reviewers performed this analysis. |
| **Effect measures** | 12 | Specify for each outcome the effect measure(s) (e.g. risk ratio, mean difference) used in the synthesis or presentation of results. | Results are presented numerically only where possible. Usually in the form of mean and standard deviation or number of readings. |
| **Synthesis methods** | 13a | Describe the processes used to decide which studies are eligible for each synthesis (e.g. tabulating the study intervention characteristics and comparing against the planned groups for each synthesis (item 5)). | Gathering the following data in tables: in human studies: author, study design, number of participants, age (years), type of intervention, control conditions, and duration of the study. For the study outcomes, the following data is extracted from all included articles: the type of material (colonic/fecal) taken for testing the composition of the microbiota, the abundance of microbial taxa or other relevant features of the microbiome, and other study findings , such as associations between changes in microbiota and metabolic markers. In animal studies, the type of diets used are also noted. |
|  | 13b | Describe any methods required to prepare the data for presentation or synthesis, such as handling of missing summary statistics, or data conversions. | In the absence of data in the work or problems with their interpretation, an attempt is made to contact the authors, unfortunately unsuccessful. Any disputes regarding the appropriateness of including or excluding a given study are resolved by discussion between the authors. |
|  | 13c | Describe any methods used to tabulate or visually display results of individual studies and syntheses. | Due to the fact that the microbiotic data do not always contain numerical data, the changes are collected in tables and illustrated with arrows. |
|  | 13d | Describe any methods used to synthesize results and provide a rationale for the choice(s). If meta-analysis is performed, describe the model(s), method(s) to identify the presence and extent of statistical heterogeneity, and software package(s) used. | The works have been collected in tables, because thanks to this, it is possible to visualize and compare the included works in a transparent way. A separation between human and animal studies is used to draw attention to the material from which the microbiota is assessed. A meta-analysis is not performed. |
|  | 13e | Describe any methods used to explore possible causes of heterogeneity among study results (e.g. subgroup analysis, meta-regression). | No meta-analysis is performed. In the case of heterogeneity of results, the biological material from which the microbiota is assessed, as well as the time and method of its collection, are considered. |
|  | 13f | Describe any sensitivity analyses conducted to assess robustness of the synthesized results. | None |
| **Reporting bias assessment** | 14 | Describe any methods used to assess risk of bias due to missing results in a synthesis (arising from reporting biases). | Due to the nature of the data, the limited number of studies, the large heterogeneity they displayed, the various designs, the various methodologies for determining microbiota, and various ways of presenting the results, it was decided to systematically summarize current evidence, rather than performing a quantitative meta-analysis. |
| **Certainty assessment** | 15 | Describe any methods used to assess certainty (or confidence) in the body of evidence for an outcome. | No meta-analysis is performed. Missing data are not presented because the authors of these papers do not reply to inquiries. |
| **RESULTS** |  |  |  |
| **Study selection** | 16a | Describe the results of the search and selection process, from the number of records identified in the search to the number of studies included in the review, ideally using a flow diagram. | Overall, 331 articles are identified, and the final analysis included seven human studies and nine animal studies from sixteen papers. The search strategy is summarized in Figure 1. |
|  | 16b | Cite studies that might appear to meet the inclusion criteria, but which are excluded, and explain why they are excluded. | None |
| **Study characteristics** | 17 | Cite each included study and present its characteristics. | This has been described in the Results section, divided into studies involving humans and animals, and divided into TRE and RF |
| **Risk of bias in studies** | 18 | Present assessments of risk of bias for each included study. | The quality of all included studies is rated “good” or “fair”. None of the studies are of “poor” quality. All animal studies are assessed as “good”. In turn, two of the human studies are rated “fair”: one of them, which is cross-sectional, gave no specific inclusion or exclusion criteria, and no exact recruitment period. The other study, which is an RCT, gave no specific randomization method is given and do not calculate a minimum sample size, despite this being necessary in this type of study. |
| **Results of individual studies** | 19 | For all outcomes, present, for each study: (a) summary statistics for each group (where appropriate) and (b) an effect estimate and its precision (e.g. confidence/credible interval), ideally using structured tables or plots. | All results are presented in the form of a description or in a table from 2 - 5 + 1 supplementary table S3. The studies are divided into those involving animals and humans. A meta-analysis has not been performed. |
| **Results of syntheses** | 20a | For each synthesis, briefly summarise the characteristics and risk of bias among contributing studies. | All results are presented in the form of a description or in a table from 2 - 5 + 1 supplementary table S3. The studies are divided into those involving animals and humans. A meta-analysis has not been performed. |
|  | 20b | Present results of all statistical syntheses conducted. If meta-analysis is done, present for each the summary estimate and its precision (e.g. confidence/credible interval) and measures of statistical heterogeneity. If comparing groups, describe the direction of the effect. | As no meta-analysis is performed, statistical analyzes are not performed. |
|  | 20c | Present results of all investigations of possible causes of heterogeneity among study results. | Taking into account the human studies in which the composition of the microbiota in the feces is examined, it is hard to determine the exact time of its collection; this undoubtedly has a large impact on the measured microbiome composition. It is worth mentioning that due to the nature of the TRE and RF, it is impossible to blind the research participants and animal caregivers. For this reason, blinding is not taken into account when assessing the quality of studies. |
|  | 20d | Present results of all sensitivity analyses conducted to assess the robustness of the synthesized results. | No meta-analysis is performed |
| **Reporting biases** | 21 | Present assessments of risk of bias due to missing results (arising from reporting biases) for each synthesis assessed. | Overall, 331 articles are identified, and the final analysis included seven human studies and nine animal studies from sixteen papers (Figure 1). The quality of all included studies is rated “good” or “fair”. None of the studies are of “poor” quality. All animal studies are assessed as “good”. In turn, two of the human studies are rated “fair”: one of them, which is cross-sectional, gave no specific inclusion or exclusion criteria, and no exact recruitment period. The other study, which is an RCT, give no specific randomization method and do not calculate a minimum sample size, despite this being necessary in this type of study. |
| **Certainty of evidence** | 22 | Present assessments of certainty (or confidence) in the body of evidence for each outcome assessed. | Due to the nature of the data, the limited number of studies, the large heterogeneity they displayed, the various designs, the various methodologies for determining microbiota, and various ways of presenting the results, it was decided to systematically summarize current evidence, rather than performing a quantitative meta-analysis. In the following sections, the studies’ findings are explicated. Differences between groups and changes within the study group before and after the intervention are reported. |
| **DISCUSSION** |  |  |  |
| **Discussion** | 23a | Provide a general interpretation of the results in the context of other evidence. | At the genus level, *Akkermansia* abundance seems to be dependent on the type of diet administered in TRE (enrichment after chow diet and decrease after HFD). On the other hand, only the RF study of Ozkul et al. among the human studies show an increase in the *Akkermansia* genus. Increase in the abundance of these bacteria seems to be extremely favorable, as it has been noted that *Akkermansia muciniphila* causes an increase in the expression of genes associated with immune responses and in the strengthening of the gut barrier function. It is also indicated that *A.muciniphila* affects glucose and lipid metabolism through the production of mucin that improves the tightness of the intestinal barrier and stimulates the immune system to secrete anti-inflammatory cytokines. |
|  | 23b | Discuss any limitations of the evidence included in the review. | The articles included in this systematic review have some limitations. In the human studies that examined fecal microbiota composition, it can be difficult to determine the exact time and method of collection (e.g., sample storage and process sterility); in many cases this leads to an inability to compare results between studies. These differences undoubtedly have a large effect on the measured microbiome composition. The same consideration applies to the examination of animal feces, as it is also not possible to collect them immediately after expulsion. Furthermore, assessing human or animal gut microbiota composition at one point in time (whether fecal or intestinal) makes it impossible to observe cyclical circadian fluctuations in the microbiota. The results of the included studies lead us to conclude that microbiota can be most accurately assessed from the intestinal contents collected at circadian termination. Another limitation of this review is that the studies it considers are based not only on different study populations (humans and animals), but also on different intervention protocols with, for example, eating windows being during the day or during night; this may cause some ambiguities and make interpretation difficult. |
|  | 23c | Discuss any limitations of the review processes used. | None |
|  | 23d | Discuss implications of the results for practice, policy, and future research. | Since only a small number of studies link changes in microbiota with improvements in metabolic or anthropometric parameters induced by the regimens studied, it is impossible to unequivocally state whether all the observed microbial and metabolic changes are actually related. Further research should thus include metagenomics and microbial and host metabolomics in their methodology to better understand the potential correlations between microbes and host health. It should be pointed out that data in this field remain limited, especially among human studies, and so it is difficult to draw meaningful conclusions about the effects of the TRE and RF on specific taxonomic groups of gut microbiota. Moreover, more precise inspection of the human diet and of time of specimen collection is necessary to better interpret studies of the gut microbiome, and to better understand the host–microbiome relationship. |
| **OTHER INFORMATION** |  |  |  |
| **Registration and protocol** | 24a | Provide registration information for the review, including register name and registration number, or state that the review is not registered. | PROSPERO registration number CRD42021278918 |
|  | 24b | Indicate where the review protocol can be accessed, or state that a protocol is not prepared. | The protocol is available from the corresponding author. |
|  | 24c | Describe and explain any amendments to information provided at registration or in the protocol. | Describe and explain any amendments to information provided at registration or in the protocol. |
| **Support** | 25 | Describe sources of financial or non-financial support for the review, and the role of the funders or sponsors in the review. | None |
| **Competing interests** | 26 | Declare any competing interests of review authors. | None |
| **Availability of data, code and other materials** | 27 | Report which of the following are publicly available and where they can be found: template data collection forms; data extracted from included studies; data used for all analyses; analytic code; any other materials used in the review. | The data search scheme, the data collection template, the results obtained, as well as the bias assessment table of the included articles are available to the public. |

#####

# PRIMSA Abstract Checklist

| **Topic** | **No.** | **Item** | **Reported?** |
| --- | --- | --- | --- |
| **TITLE** |  |  |  |
| **Title** | 1 | Identify the report as a systematic review. | Yes |
| **BACKGROUND** |  |  |  |
| **Objectives** | 2 | Provide an explicit statement of the main objective(s) or question(s) the review addresses. | Yes |
| **METHODS** |  |  |  |
| **Eligibility criteria** | 3 | Specify the inclusion and exclusion criteria for the review. | Yes |
| **Information sources** | 4 | Specify the information sources (e.g. databases, registers) used to identify studies and the date when each is last searched. | Yes |
| **Risk of bias** | 5 | Specify the methods used to assess risk of bias in the included studies. | Yes |
| **Synthesis of results** | 6 | Specify the methods used to present and synthesize results. | Yes |
| **RESULTS** |  |  |  |
| **Included studies** | 7 | Give the total number of included studies and participants and summarise relevant characteristics of studies. | Yes |
| **Synthesis of results** | 8 | Present results for main outcomes, preferably indicating the number of included studies and participants for each. If meta-analysis is done, report the summary estimate and confidence/credible interval. If comparing groups, indicate the direction of the effect (i.e. which group is favoured). | Yes |
| **DISCUSSION** |  |  |  |
| **Limitations of evidence** | 9 | Provide a brief summary of the limitations of the evidence included in the review (e.g. study risk of bias, inconsistency and imprecision). | Yes |
| **Interpretation** | 10 | Provide a general interpretation of the results and important implications. | Yes |
| **OTHER** |  |  |  |
| **Funding** | 11 | Specify the primary source of funding for the review. | Yes |
| **Registration** | 12 | Provide the register name and registration number. | Yes |

*From:* Page MJ, McKenzie JE, Bossuyt PM, Boutron I, Hoffmann TC, Mulrow CD, et al. The PRISMA 2020 statement: an updated guideline for reporting systematic reviews. MetaArXiv. 2020, September 14. DOI: 10.31222/osf.io/v7gm2. For more information, visit: <www.prisma-statement.org>
