# Supplementary material for: The effects of time-restricted eating and Ramadan fasting on gut microbiota composition: a systematic review of human and animal studies
Source: Nutr Rev. 2023 Aug 1;82(6):777–93. doi: 10.1093/nutrit/nuad093 (PMC11082590; doi:10.1093/nutrit/nuad093)
Supplement: nuad093_Supplementary_Data [file nuad093_supplementary_data.zip › nuad093_Supplementary_Data/26.06 Supplementary tables Nut Rev.docx]

Supplementary Table S1. Evaluation of the quality of animal studies

| **Item** | **Type of bias** | **Domain** | **Description of domain** | **Review authors judgment** | **Signalling questions** | **Hu et al. 2018**^1^ | **Ye et al. 2020**^2^ | **Zarrinpar et al. 2014**^3^ | **Li et al. 2020** ^4^ | **Van der Merwe et al. 2020**^5^ | **Palomba et al. 2021** ^6^ | **He et al. 2021**^7^ | **Su et al. 2022**^8^ | **Machado et al. 2022**^9^ |
| --- | --- | --- | --- | --- | --- | --- | --- | --- | --- | --- | --- | --- | --- | --- |
| 1 | Selection bias | Sequence generation | Describe the methods used, if any, to generate the allocation sequence in sufficient detail to allow an assessment whether it should produce comparable groups. | Was the allocation sequence adequately generated and applied? (*) | Did the investigators describe a random component in the sequence generation process such as Referring to a random number table; Using a computer random number generator. | Unclear | Unclear | Unclear | Unclear | Unclear | No | Unclear | Unclear | No |
| 2 | Selection bias | Baseline characteristics | Describe all the possible prognostic factors or animal characteristics, if any, that are compared in order to judge whether or not intervention and control groups were similar at the start of the experiment. | Were the groups similar at baseline or were they adjusted for confounders in the analysis? | Was the distribution of relevant baseline characteristics balanced for the intervention and control groups? | Yes | Yes | Yes | Yes | Yes | Yes | Yes | No | Yes |
|  |  |  |  |  | If relevant, did the investigators adequately adjust for unequal distribution of some relevant baseline characteristics in the analysis? | X | X | X | X | X | X | X | Unclear | X |
|  |  |  |  |  | Was the timing of disease induction adequate? | X | X | X | X | X | X | X | X | X |
| 3 | Selection bias | Allocation concealment | Describe the method used to conceal the allocation sequence in sufficient detail to determine whether intervention allocations could have been foreseen before or during enrolment. | Was the allocation adequately concealed? (*) | Could the investigator allocating the animals to intervention or control group not foresee assignment due to one of the following or equivalent methods? | Unclear | Unclear | Unclear | Unclear | Unclear | No | Unclear | Unclear | No |
| 4 | Performance bias | Random housing | Describe all measures used, if any, to house the animals randomly within the animal room. | Were the animals randomly housed during the experiment? | Did the authors randomly place the cages or animals within the animal room/facility? | Unclear | Unclear | Unclear | Unclear | Unclear | Unclear | Unclear | Unclear | Unclear |
|  |  |  |  |  | Is it unlikely that the outcome or the outcome measurement was influenced by not randomly housing the animals? | Yes | Yes | Yes | Yes | Yes | Yes | Yes | Yes | Yes |
| 5 | Performance bias | Blinding | Describe all measures used, if any, to blind trial caregivers and researchers from knowing which intervention each animal received. Provide any information relating to whether the intended blinding was effective. | Were the caregivers and/or investigators blinded from knowledge which intervention each animal received during the experiment? | Was blinding of caregivers and investigators ensured, and was it unlikely that their blinding could have been broken? | No | No | No | No | No | No | No | No | No |
| 6 | Detection bias | Random outcome assessment | Describe whether or not animals were selected at random for outcome assessment, and which methods to select the animals, if any, were used. | Were animals selected at random for outcome assessment? | Did the investigators randomly pick an animal during outcome assessment, or did they use a random component in the sequence generation for outcome assessment? | Unclear | Unclear | Unclear | Unclear | Unclear | Yes | Unclear | Unclear | Unclear |
| 7 | Detection bias | Blinding | Describe all measures used, if any, to blind outcome assessors from knowing which intervention each animal received. Provide any information relating to whether the intended blinding was effective. | Was the outcome assessor blinded? | Was blinding of the outcome assessor ensured, and was it unlikely that blinding could have been broken? | No | No | No | No | No | Yes | No | No | No |
|  |  |  |  |  | Was the outcome assessor not blinded, but do review authors judge that the outcome is not likely to be influenced by lack of blinding? | Yes | Yes | Yes | Yes | Yes | X | Yes | Yes | Yes |
| 8 | Attrition bias | Incomplete outcome data | Describe the completeness of outcome data for each main outcome, including attrition and exclusions from the analysis. State whether attrition and exclusions were reported, the numbers in each intervention group (compared with total randomized animals), reasons for attrition or exclusions, and any re-inclusions in analyses for the review. | Were incomplete outcome data adequately addressed? (*) | Were all animals included in the analysis? | Yes | Yes | Yes | No | No | Yes | Yes | Yes | Yes |
|  |  |  |  |  | Were the reasons for missing outcome data unlikely to be related to true outcome? (e.g., technical failure) | X | X | X | X | X | X | X | X | X |
|  |  |  |  |  | Are missing outcome data balanced in numbers across intervention groups, with similar reasons for missing data across groups? | X | X | X | X | X | X | X | X | X |
|  |  |  |  |  | Are missing outcome data imputed using appropriate methods? | X | X | X | X | X | X | X | X | X |
| 9 | Reporting bias | Selective outcome reporting | State how selective outcome reporting was examined and what was found. | Are reports of the study free of selective outcome reporting? (*) | Was the study protocol available and were all of the study’s pre-specified primary and secondary outcomes reported in the current manuscript? | Yes | Yes | Yes | Yes | Yes | Yes | Yes | Yes | Yes |
|  |  |  |  |  | Was the study protocol not available, but was it clear that the published report included all expected outcomes (i.e. comparing methods and results section)? | X | X | X | X | X | X | X | X | X |
| 10 | Other | Other sources of bias | State any important concerns about bias not covered by other domains in the tool. | Was the study apparently free of other problems that could result in high risk of bias? (*) | Was the study free of contamination (pooling drugs)? | Yes | Yes | Yes | Yes | Yes | Yes | Yes | Yes | Yes |
|  |  |  |  |  | Was the study free of inappropriate influence of funders? | Yes | Yes | Yes | Yes | Yes | Yes | Yes | Yes | Yes |
|  |  | Signalling questions | <https://www.ncbi.nlm.nih.gov/pmc/articles/PMC4230647/table/T3/?report=objectonly> |  | Was the study free of unit of analysis errors? | Yes | Yes | Yes | Yes | Yes | Yes | Yes | Yes | Yes |
|  |  |  |  |  | Were design-specific risks of bias absent? | Yes | Yes | Yes | Yes | Yes | Yes | Yes | Yes | Yes |
|  |  |  |  |  | Were new animals added to the control and experimental groups to replace drop-outs from the original population? | X | X | X | No | No | X | X | X | X |
| **Global** | | | | | | Good | Good | Good | Good | Good | Good | Good | Good | Good |

Supplementary Table S2. Evaluation of the quality of human studies

| **Quality Assessment Tool for Observational Cohort and Cross-Sectional Studies** | Ozkul et al. 2020^10^ | Su et al. 2021^11^ | Ali et al. 2021^12^ | Zeb et al. 2020^13^ | **Quality Assessment Tool for Before-After (Pre-Post) Studies With No Control Group** | Gabel et al. 2020^14^ | **Quality Assessment of Controlled Intervention Studies** | Zeb et al. 2020^15^ | Xie et al. 2022^16^ |
| --- | --- | --- | --- | --- | --- | --- | --- | --- | --- |
| 1. Was the research question or objective in this paper clearly stated? | Yes | Yes | Yes | Yes | 1. Was the study question or objective clearly stated? | Yes | 1. Was the study described as randomized, a randomized trial, a randomized clinical trial, or an RCT? | Yes | Yes |
| 2. Was the study population clearly specified and defined? | Yes | Yes | Yes | Yes | 2. Were eligibility/selection criteria for the study population prespecified and clearly described? | Yes | 2. Was the method of randomization adequate (i.e., use of randomly generated assignment)? | NR | Yes |
| 3. Was the participation rate of eligible persons at least 50%? | Yes | Yes | Yes | Yes | 3. Were the participants in the study representative of those who would be eligible for the test/service/intervention in the general or clinical population of interest? | Yes | 3. Was the treatment allocation concealed (so that assignments could not be predicted)? | X | X |
| 4. Were all the subjects selected or recruited from the same or similar populations (including the same time period)? Were inclusion and exclusion criteria for being in the study prespecified and applied uniformly to all participants? | Yes | Yes | Yes | No | 4. Were all eligible participants that met the prespecified entry criteria enrolled? | Yes | 4. Were study participants and providers blinded to treatment group assignment? | No | No |
| 5. Was a sample size justification, power description, or variance and effect estimates provided? | No | No | No | No | 5. Was the sample size sufficiently large to provide confidence in the findings? | Yes | 5. Were the people assessing the outcomes blinded to the participants' group assignments? | Yes | Yes |
| 6. For the analyses in this paper, were the exposure(s) of interest measured prior to the outcome(s) being measured? | NA | NA | NA | NA | 6. Was the test/service/intervention clearly described and delivered consistently across the study population? | Yes | 6. Were the groups similar at baseline on important characteristics that could affect outcomes (e.g., demographics, risk factors, co-morbid conditions)? | Yes | Yes |
| 7. Was the timeframe sufficient so that one could reasonably expect to see an association between exposure and outcome if it existed? | Yes | Yes | Yes | Yes | 7. Were the outcome measures prespecified, clearly defined, valid, reliable, and assessed consistently across all study participants? | Yes | 7. Was the overall drop-out rate from the study at endpoint 20% or lower of the number allocated to treatment? | Yes | Yes |
| 8. For exposures that can vary in amount or level, did the study examine different levels of the exposure as related to the outcome (e.g., categories of exposure, or exposure measured as continuous variable)? | NA | NA | NA | NA | 8. Were the people assessing the outcomes blinded to the participants' exposures/interventions? | No | 8. Was the differential drop-out rate (between treatment groups) at endpoint 15 percentage points or lower? | Yes | Yes |
| 9. Were the exposure measures (independent variables) clearly defined, valid, reliable, and implemented consistently across all study participants? | NA | NA | NA | NA | 9. Was the loss to follow-up after baseline 20% or less? Were those lost to follow-up accounted for in the analysis? | Yes | 9. Was there high adherence to the intervention protocols for each treatment group? | NR | NR |
| 10. Was the exposure(s) assessed more than once over time? | NA | NA | NA | NA | 10. Did the statistical methods examine changes in outcome measures from before to after the intervention? Were statistical tests done that provided p values for the pre-to-post changes? | Yes | 10. Were other interventions avoided or similar in the groups (e.g., similar background treatments)? | Yes | Yes |
| 11. Were the outcome measures (dependent variables) clearly defined, valid, reliable, and implemented consistently across all study participants? | Yes | Yes | Yes | Yes | 11. Were outcome measures of interest taken multiple times before the intervention and multiple times after the intervention (i.e., did they use an interrupted time-series design)? | No | 11. Were outcomes assessed using valid and reliable measures, implemented consistently across all study participants? | Yes | Yes |
| 12. Were the outcome assessors blinded to the exposure status of participants? | No | No | No | No | 12. If the intervention was conducted at a group level (e.g., a whole hospital, a community, etc.) did the statistical analysis take into account the use of individual-level data to determine effects at the group level? | CD | 12. Did the authors report that the sample size was sufficiently large to be able to detect a difference in the main outcome between groups with at least 80% power? | NR | Yes |
| 13. Was loss to follow-up after baseline 20% or less? | NR | NR | NR | NR |  |  | 13. Were outcomes reported or subgroups analyzed prespecified (i.e., identified before analyses were conducted)? | No | No |
| 14. Were key potential confounding variables measured and adjusted statistically for their impact on the relationship between exposure(s) and outcome(s)? | Yes | Yes | NR | Yes |  |  | 14. Were all randomized participants analyzed in the group to which they were originally assigned, i.e., did they use an intention-to-treat analysis? | Yes | Yes |
| **Global** | **Good** | **Good** | **Good** | **Fair** |  | **Good** |  | **Fair** | **Good** |

Supplementary Table S3. Effects of TRE regimen at the phylum and genus level, and in alpha and beta diversity, in animal studies.

| **Source** | **Source of microbiota** | | | **Comparison** | **Phylum – differences between groups** | | **Phylum – differences between different phases** | | | **Genus (differences between groups)** | **Genus – differences between different phases** | | **alpha-diversity** | **beta-diversity** |
| --- | --- | --- | --- | --- | --- | --- | --- | --- | --- | --- | --- | --- | --- | --- |
| **Circadian termination** | | | | | | | | | | | | | | |
| Zarrinpar et al. 2014 ^3^ | Cecum sample; every 4 hours over 24 h (ZT1, 5, 9, 13, 17, 21) | | | HFD TRE vs. HFD AL | *↔Firmicutes*  *↔Bacteroidetes*  *↔Veruccomicrobia* | | NR | | | ↗ *Oscillibacter  (0.40 ± 0.08% vs. 0.13 ± 0.04%)*  ↘ *Lactobacillus*  (0.97 ± 0.49% vs. 3.70 ± 1.01%) | ↓ *Lactococcus* (light phase)  (2.66 ± 0.84% vs. 0.45 ± 0.16%,)  ↓ *Lactobacillus* (dark phase)  (3.62% ± 1.49% vs. 0.06% ± 0.04%) | | ↔ | √ |
| Zarrinpar et al. 2014 ^3^ | Cecum sample; every 4 hours over 24 h (ZT1, 5, 9, 13, 17, 21) | | | HFD TRE vs. CD AL | *↔Firmicutes*  *↔Bacteroidetes*  *↔Veruccomicrobia* | | NR | | | ↘ *Lactobacillus*  (0.97 ± 0.49% vs. 3.70 ± 1.01%) | NR | | ↓ | √ |
| Ye et al. 2020 ^2^ | rectal samples; ZT0, ZT8, ZT12 & ZT20 | | | HFD TRE vs. HFD AL | ↘*Firmicutes*  *(58.04 ± 9.33% vs. 34.10 ± 13.49%)*  ↗ *Bacteroidetes (39.28 ± 17.08% vs. 27.02 ± 13.06%)*  *↔ Proteobacteria*  *↔ Actinobacteria* | | Light phase | ZT0  ZT8 | ↔ *Firmicutes*  ↔ *Bacteroidetes*  ↔ *Firmicutes*  ↔ *Bacteroidetes* | NR | NR | | ↔ | x |
|  |  |  |  |  |  |  | Dark phase | ZT12  ZT20 | ↔ *Firmicutes*  ↔ *Bacteroidetes*  ↓ *Firmicutes*  (35.04 ± 9.38% vs. 52.77 ± 7.73%)  ↑*Bacteroidetes*  (57.58 ± 10.77% vs. 29.27 ± 11.56%) |  |  |  |  |  |
| Ye et al. 2020 ^2^ | rectal samples; ZT0, ZT8, ZT12 & ZT20 | | | HFD TRE vs. CD AL | ↗ *Firmicutes*  *(47.89 ± 12.86% vs. 34.1 ± 13.49%)*  ↘*Bacteroidetes*  *(39.28 ± 17.08% vs. 61.34 ± 12.99%)*    ↗ *Proteobacteria (9.471 ± 5.918 vs. 2.34 ± 1.38%)*  *↔ Actinobacteria* | | Light phase | ZT0  ZT8 | ↑ *Firmicutes*  ↓ *Bacteroidetes*  ↔ *Firmicutes*  ↔ *Bacteroidetes*  ↑ *Firmicutes*  ↓ *Bacteroidetes*  ↔ *Bacteroidetes*  ↔ *Firmicutes* | NR | NR | | ↔ | √ |
|  |  |  |  |  |  |  | Dark phase | ZT12  ZT20 |  |  |  |  |  |  |
| He et al. 2021^7^ | Cecal samples after 4 weeks of LD | | | LD RF-like vs. LD AL | NR | | *Light phase* | ZT0  ZT4  ZT8 | *↑ Firmicutes*  *↓ Veruccomicrobia*  *↔ Deferribacteres*  *↔ Actinobacteria*  *↔ Proteobacteria*  *↔ Bacteroidetes*  *↑ Proteobacteria*  *↔ Firmicutes*  *↔ Deferribacteres*  *↔ Veruccomicrobia*  *↔ Actinobacteria*  *↔ Bacteroidetes*  *↑ Firmicutes*  *↓Bacteroidetes*  *↑ Proteobacteria*  *↑ Actinobacteria*  *↔ Deferribacteres*  *↔ Veruccomicrobia* | NR | NR | | NR | √ |
|  |  |  |  |  |  |  | Dark phase | ZT12  ZT16  ZT20 | *↑ Proteobacteria*  *↓ Actinobacteria*  *↔ Deferribacteres*  *↔ Veruccomicrobia*  *↔ Firmicutes*  *↔ Bacteroidetes*  *↑ Bacteroidetes*  *↑ Actinobacteria*  *↔ Deferribacteres*  *↔ Veruccomicrobia*  *↔ Firmicutes*  *↔ Proteobacteria*  *↔ Deferribacteres*  *↔ Veruccomicrobia*  *↔ Firmicutes*  *↔ Actinobacteria*  *↔ Proteobacteria*  *↔ Bacteroidetes* |  |  |  |  |  |
| Machado et al. 2022 ^9^ | | Ileal samples;  ZT1, ZT4, ZT9, ZT13, ZT17, ZT21;  after HFD | HFD TRE vs. HFD AL | | | ↔ *Bacteroidetes* |  | | | *↑ Enterococcus*  *↑ Staphylococcus*  *↑ Lactococcus*  *↑ Proteiniphilum*  *↑ Bacillus*  *↑ Colidextribacter*  *↑ Blautia*  *↑ Tuzzerella*  *↑ Angelakisella*  *↑ Helicobacter*  *↑ Parasutterella*  *↑ Acetatifactor*  *↑ Oscillospira*  *↓ Stenotrophomonas*  *↓ Eryspilatoclostridium*  *↓ Muribaculaceae*  *↓ A2*  *↓ Oscillospirales*  *↓ Ruminococcus*  *↓ Lachnoclostridium*  *↓ Turicibacter*  *↓ Bifidobacterium*  *↓ Pseudonocardiaceae*  *↓ Alistipes*  *↓ Dubosiella*  *↓ Akkermansia* | Light phase | *↑ Ruminococcaceae/Lactococcus*  *↓ Turicibacter/Enterococcus*  *↑Enterococcus/Lactococcus* | ↔ (Shannon Index)  ↔ (Faith`s PD) | √ |
|  |  |  |  |  |  |  |  |  |  |  | Dark phase | *↔ Ruminococcaceae/Lactococcus*  *↓ Turicibacter/Enterococcus*  *↔ Enterococcus/Lactococcus*  ZT13 *↑ Staphylococcus* |  |  |
| Machado et al. 2022^9^ | | Ileal samples;  ZT1, ZT4, ZT9, ZT13, ZT17, ZT21;  after HFD | HFD TRE vs. CD AL | | | ↘*Bacteroidetes* |  | | | *↑ Stenotrophomonas*  *↑ Staphylococcus*  *↑ Leuconostoc*  *↑ Colidextribacter*  *↑ Cutibacterium*  *↑ Harryflintia*  *↑ Blautia*  *↑ Erysipelatoclostridium*  *↑ Pseudonocardiaceae*  *↑ Proteiniphilum*  *↑ Enterococcus*  *↑ Tuzzerella*  *↑ Lactococcus*  *↓ Ruminococcaceae*  *↓ Turicibacter*  *↓ Lachnoclostridium*  *↓ Ruminococcus*  *↓ Dubosiella*  *↓ Weisella*  *↓ Butyricoccus*  *↓ Uncultured*  *↓ Faecalibaculum*  *↓ ASF356*  *↓ Muribaculaceae*  *↓ Monoglobus*  *↓ Alistipes* | *Light phase* | *↓ Ruminococcaceae/Lactococcus*  *↓ Turicibacter/Enterococcus*  *↔ Enterococcus/Lactococcus* | ↔ (Shannon Index)  ↓ (Faith`s PD) | √ |
|  |  |  |  |  |  |  |  |  |  |  | *Dark phase* | *↓ Ruminococcaceae/Lactococcus*  *↓ Turicibacter/Enterococcus*  *↓ Enterococcus/Lactococcus*  ZT13 *↔ Staphylococcus* |  |  |
| Machado et al. 2022 ^9^ | | Cecal samples;  ZT1, ZT4, ZT9, ZT13, ZT17, ZT21;  after HFD | HFD TRE vs. HFD AL | | | NR | NR | | | NR | NR | | ↔ (Shannon Index)  ↔ (Faith`s PD) | √ |
| Machado et al. 2022^9^ | | Cecal samples;  ZT1, ZT4, ZT9, ZT13, ZT17, ZT21;  after HFD | HFD TRE vs. CD AL | | | NR | NR | | | NR | NR | | ↔ (Shannon Index)  ↓ (Faith`s PD) | √ |
| **Termination at one ZT point** | | | | | | | | | | | | | | |
| Hu et al. 2018^1^ | Cecal samples; ZT21; after CD | | | CD TRE vs. CD AL | ↗ *Firmicutes*  ↘ *Bacteroidetes* | |  | | | ↗ *Lactobacillus* ↗ *Roseburia*  ↘ *Staphylococcus* |  | | ↔ | √ |
| Li et al. 2020  (16 hours of fasting)^4^ | Fecal sample; day 30 & day 60; after CD | | | CD TRE vs. CD AL | NR | |  | | | ↗ *Akkermansia*  ↘ *Alistipes* |  | | ↔ | √ |
| Li et al. 2020  (12 hours of fasting) ^4^ | Fecal sample; day 30 & day 60; after CD | | | CD TRE vs. CD AL | No taxonomic differences | |  | | | No taxonomic differences |  | | ↔ | √ |
| Li et al. 2020  (20 hours of fasting) ^4^ | Fecal sample; day 30 & day 60; after CD | | | CD TRE vs. CD AL | No taxonomic differences | |  | | | No taxonomic differences |  | | ↔ | √ |
| Van der Merwe et al. 2020^5^ | Fecal samples; after 6 wk of HFD (T0) & again at 3 wk (T1) & 7 wk (T2) | | | HFD TRE vs. HFD AL | NR | |  | | | ↗ *Ruminococcus,* ↗ *Mucispirillum,* ↗ *Desulfovibrio,* ↗ *Coprococcus,*  ↗ *Lactococcus*  ↗ *Enterococcus* |  | | ↑ | x |
|  | Cecal samples; after HFD | | | HFD TRE vs. HFD AL | ↗ Verrucomicrobia (6%)  (unknown significance) | |  | | | ↗ *Lactococcus*  ↗ *Akkermansia*  ↘ *Bilophila* |  | | NR | x |
| Palomba et al. 2021 ^6^ | Fecal sample; after 48 wk of CD | | | CD TRE vs. CD AL | NR | |  | | | ↗ *Akkermansia (4327 vs 15),* ↗ *Anaerovorax (489 vs 210),*  ↗ *Marvinbryantia (76 vs 20),*  ↘ *Adlercreutzia (*546 vs 236),  ↘ *Enterorhabdus (1007 vs 422),*  ↘ *Bilophila (52 vs 21),*  ↘ *Lactococcus (386 vs 46),*  ↘ *Romboutsia (2221 vs 510),*  ↘ *Ruminiclostridium (235 vs 94),*  ↘ *Ruminococcus (11948 vs 2979)*  ↘ *Turicibacter* (576 vs 167)  ↘ *Candidatus Soleaferrea (*42 vs 99)  *↔ Butyricimonas*  *↔ Lachnoclostridium*  *↔ Rothia*  *↔ Monoglobus*  *↔ Elusimicrobium*  *↔ Blautia*  *↔ Roseburia*  *↔ Veillonella*  *↔ Papillibacter*  *↔ Parasutterella*  *↔ Parabacteroides*  *↔ Intestinimonas*  *↔ Alistipes*  *↔ Colidextribacter*  *↔ Helicobacter*  *↔ Pygmaiobacter*  *↔ Paludicola*  *↔ Phascolarctobacterium*  *↔ Methanosphaera*  *↔ Bacteroides*  *↔ Tuzzerella*  *↔ Treponema*  *↔ Prevotella*  *↔ Oscillibacter*  *↔ Alloprevotella*  *↔ Negativibacillus*  *↔ Angelakisella*  *↔ Streptococcus*  *↔ Desulfovibrio*  *↔ Odoribacter*  *↔ Lactobacillus*  *↔ Candidatus*  *↔ Saccharimonas* |  | | NR | NR |
| Su et al. 2022^8^ | Fecal sample; day 0 & day 30; after CD | | | CD RF-like vs. CD AL | ↗ *Firmicutes (52.79 ± 7.48 vs.* *67.53 ± 4.84)*  ↘ *Bacteroidetes (38.79 ± 4.93 vs. 24.39 ± 6.12)* | |  | | | NR |  | | ↔ | √ |

↓ a significant decrease; ↔ there was non-significant effect; ↑ a significant increase; ↗ - enrichment in comparison to control group; ↘ - depletion in comparison to control group; √- changes in beta diversity, x – no changes in beta diversity. In the case of two control groups (Ye, Zarrinpar), the given group in relation to which a change was assesed is shown in brackets. If there is no bracket for a given change, it means that the change applies to both control groups.

**Abbreviations: TRE** – time restricted feeding, **HFD** – high fat diet, **CD** – chow diet, **LD** – lithogenic diet, **ZT** - zeitgeber time, **NR** – not reported

**References**:

1. Hu D, Mao Y, Xu G, Liao W, Yang H, Zhang H. Gut flora shift caused by time-restricted feeding might protect the host from metabolic syndrome, inflammatory bowel disease and colorectal cancer. *Transl Cancer Res*. 2018;7(5):1282-1289. doi:10.21037/tcr.2018.10.18

2. Ye Y, Xu H, Xie Z, et al. Time-Restricted Feeding Reduces the Detrimental Effects of a High-Fat Diet, Possibly by Modulating the Circadian Rhythm of Hepatic Lipid Metabolism and Gut Microbiota. *Front Nutr*. 2020;7:596285. doi:10.3389/fnut.2020.596285

3. Zarrinpar A, Chaix A, Yooseph S, Panda S. Diet and Feeding Pattern Affect the Diurnal Dynamics of the Gut Microbiome. *Cell Metab*. 2014;20(6):1006-1017. doi:10.1016/j.cmet.2014.11.008

4. Li L, Su Y, Li F, et al. The effects of daily fasting hours on shaping gut microbiota in mice. *BMC Microbiol*. 2020;20(1):65. doi:10.1186/s12866-020-01754-2

5. van der Merwe M, Sharma S, Caldwell JL, et al. Time of Feeding Alters Obesity-Associated Parameters and Gut Bacterial Communities, but Not Fungal Populations, in C57BL/6 Male Mice. *Curr Dev Nutr*. 2020;4(2):nzz145. doi:10.1093/cdn/nzz145

6. Palomba A. Time-restricted feeding induces Lactobacillus- and Akkermansia-specific functional changes in the rat fecal microbiota. *Npj Bioﬁlms Microbiomes*. Published online 2021:10.

7. He C, Shen W, Chen C, et al. Circadian Rhythm Disruption Influenced Hepatic Lipid Metabolism, Gut Microbiota and Promoted Cholesterol Gallstone Formation in Mice. *Front Endocrinol*. 2021;12:723918. doi:10.3389/fendo.2021.723918

8. Su J, Li F, Wang Y, et al. Investigating Ramadan Like Fasting Effects on the Gut Microbiome in BALB/c Mice. *Front Nutr*. 2022;9. doi:10.3389/fnut.2022.832757

9. Machado ACD, Brown SD, Lingaraju A, et al. Diet and feeding pattern modulate diurnal dynamics of the ileal microbiome and transcriptome. *Cell Rep*. 2022;40(1):111008. doi:10.1016/j.celrep.2022.111008

10. Ozkul C, Yalinay M, Karakan T. Structural changes in gut microbiome after Ramadan fasting: a pilot study. *Benef Microbes*. 2020;11(3):227-233. doi:10.3920/BM2019.0039

11. Su J, Wang Y, Zhang X, et al. Remodeling of the gut microbiome during Ramadan-associated intermittent fasting. *Am J Clin Nutr*. 2021;113(5):1332-1342. doi:10.1093/ajcn/nqaa388

12. Ali I, Liu K, Long D, et al. Ramadan Fasting Leads to Shifts in Human Gut Microbiota Structured by Dietary Composition. *Front Microbiol*. 2021;12:642999. doi:10.3389/fmicb.2021.642999

13. Zeb F, Wu X, Chen L, et al. Time-restricted feeding is associated with changes in human gut microbiota related to nutrient intake. *Nutr Burbank Los Angel Cty Calif*. 2020;78:110797. doi:10.1016/j.nut.2020.110797

14. Gabel K, Marcell J, Cares K, et al. Effect of time restricted feeding on the gut microbiome in adults with obesity: A pilot study. *Nutr Health*. 2020;26(2):79-85. doi:10.1177/0260106020910907

15. Zeb F, Wu X, Chen L, et al. Effect of time-restricted feeding on metabolic risk and circadian rhythm associated with gut microbiome in healthy males. *Br J Nutr*. 2020;123(11):1216-1226. doi:10.1017/S0007114519003428

16. Xie Z, Sun Y, Ye Y, et al. Randomized controlled trial for time-restricted eating in healthy volunteers without obesity. *Nat Commun*. 2022;13(1):1003. doi:10.1038/s41467-022-28662-5
