# Supplementary material for: The effects of time-restricted eating and Ramadan fasting on gut microbiota composition: a systematic review of human and animal studies
Source: Nutr Rev. 2023 Aug 1;82(6):777–93. doi: 10.1093/nutrit/nuad093 (PMC11082590; doi:10.1093/nutrit/nuad093)
Supplement: nuad093_Supplementary_Data [file nuad093_supplementary_data.zip › nuad093_Supplementary_Data/26.06 Cover_letter.doc]

Joanna Bajerska, Assoc. Prof.	Poznań, 26.06.2023

Department of Human Nutrition and Dietetics

Poznan University of Life Sciences

Wojska Polskiego 31, 60-624 Poznan, Poland

tel.+48 61 846 60 56, fax: +48 61 848 73 32

e-mail: joanna.bajerska@up.poznan.pl

Editor-in-Chief

Douglas Taren, PhD

Dear Editor,

Thank you very much for accepting our manuscript entitled **The effects of time-restricted eating and Ramadan fasting on gut microbiota composition: A systematic review of human and animal studies**, authored by Joanna Maria Pieczyńska-Zając, Anna Malinowska, Karolina Łagowska, Natalia Leciejewska and Joanna Bajerska. We resubmit the manuscript once the editorial changes have been made according to the Editors remarks.

Yours faithfully

/Joanna Bajerska/
