# Supplementary material for: The effects of time-restricted eating and Ramadan fasting on gut microbiota composition: a systematic review of human and animal studies
Source: Nutr Rev. 2023 Aug 1;82(6):777–93. doi: 10.1093/nutrit/nuad093 (PMC11082590; doi:10.1093/nutrit/nuad093)
Supplement: nuad093_Supplementary_Data [file nuad093_supplementary_data.zip › nuad093_Supplementary_Data/Answers to Editor.docx]

We would like to thank the Editor for their detailed comments and suggestions for the manuscript. After completion of the suggested edits, the revised manuscript has benefitted from an improvement in the overall presentation and clarity. Below, you will find a point-by-point description of how each comment was addressed in the manuscript.

**Editor**

Dear Prof. Bajerska:
Congratulations on the recent acceptance of the above-referenced article for publication in Nutrition Reviews. While reviewing the files, a few areas were identified that require correction or clarification prior to formatting and copyediting. The manuscript is consequently being returned to your author center with the request that the following modifications be made:

1. Please upload a clean copy of the final manuscript that includes no tracked changes or highlighting.

**Authors’ response:** All highlighting and tracked changes have been removed.

2. For your systematic review, please present the PICOS criteria (participants, interventions, comparisons, outcomes, and study design) used to define the research question as Table 1 and cite the table at an appropriate place in the text.

**Authors’ response:** PICOS criteria has been presented in Table 1 and cited in the text.

3. Please provide a list of included figures immediately after your references.

**Authors’ response:** The list of figures has been added.

4. Please provide a list of included tables immediately after your references.

**Authors’ response:** The list of tables has been added.

5. For tables or figures in which references are cited, please additionally cite the reference numbers directly after the first mention of the table or figure in the text. When doing so, please ensure that the reference numbers fit within the numerical sequence in the manuscript.

**Authors’ response:** The references have been added after the first mention of each table in the manuscript and their fit the sequence in the whole manuscript.

6. Personal pronouns should be avoided. Please rephrase sentences containing "we" and "our" to avoid their use.

**Authors’ response:** Personal pronouns have been removed and the sentences have been rephrased.

7. Please include a section for "Author contributions" in the acknowledgements. When doing so, please make sure all authors are included and that they meet the criteria for authorship established in the journal’s Instructions to Authors. Their role(s) in the conception, design, data collection or analysis, writing or revision, and final reading and approval of the manuscript should be defined in this section. <https://academic.oup.com/nutritionreviews/pages/General_Instructions#Authorship%20and%20originality>.

**Authors’ response:** The Author contributions section has been added. Also the funding section and declaration of interest have been added to the acknowledgements.

8. For Supporting Information items that contain references, please insert a separate reference list underneath the table or figure that lists all of the references that the figure contains. These references should be preceded by the letter "S" to distinguish them from the references in the main text, e.g. S1, S2, S3.

**Authors’ response:** A separate list of references has been added. The letter S has also been added to distinguish the tables.
